# Myeloid cell-associated aromatic amino acid metabolism facilitates CNS myelin regeneration

**DOI:** 10.1038/s41536-023-00345-9

**Published:** 2024-01-02

**Authors:** Jingwen Hu, George S. Melchor, Dimitrios Ladakis, Joan Reger, Hee Won Kim, Kelly A. Chamberlain, Nataliia V. Shults, Helena C. Oft, Victoria N. Smith, Lauren M. Rosko, Erqiu Li, Maryna Baydyuk, Meng-Meng Fu, Pavan Bhargava, Jeffrey K. Huang

**Affiliations:** 1 Department of Biology, Georgetown University, Washington, DC 20007 USA; 2 Interdisciplinary Program in Neuroscience, Georgetown University, Washington, DC 20007 USA; 3 Division of Neuroimmunology and Neurological Infections, Johns Hopkins University, Baltimore, MD 21287 USA; 4 National Institute of Neurological Disorders and Stroke (NINDS), National Institutes of Health (NIH), Bethesda, MD 20892 USA

**Keywords:** Regeneration and repair in the nervous system, Multiple sclerosis

## Abstract

Regulation of myeloid cell activity is critical for successful myelin regeneration (remyelination) in demyelinating diseases, such as multiple sclerosis (MS). Here, we show aromatic alpha-keto acids (AKAs) generated from the amino acid oxidase, interleukin-4 induced 1 (IL4I1), promote efficient remyelination in mouse models of MS. During remyelination, myeloid cells upregulated the expression of IL4I1. Conditionally knocking out IL4I1 in myeloid cells impaired remyelination efficiency. Mice lacking IL4I1 expression exhibited a reduction in the AKAs, phenylpyruvate, indole-3-pyruvate, and 4-hydroxyphenylpyruvate, in remyelinating lesions. Decreased AKA levels were also observed in people with MS, particularly in the progressive phase when remyelination is impaired. Oral administration of AKAs modulated myeloid cell-associated inflammation, promoted oligodendrocyte maturation, and enhanced remyelination in mice with focal demyelinated lesions. Transcriptomic analysis revealed AKA treatment induced a shift in metabolic pathways in myeloid cells and upregulated aryl hydrocarbon receptor activity in lesions. Our results suggest myeloid cell-associated aromatic amino acid metabolism via IL4I1 produces AKAs in demyelinated lesions to enable efficient remyelination. Increasing AKA levels or targeting related pathways may serve as a strategy to facilitate the regeneration of myelin in inflammatory demyelinating conditions.

## Introduction

Multiple sclerosis (MS) is a neuroinflammatory disease where myelin sheaths from multiple regions of the central nervous system (CNS) are destroyed^1^. Spontaneous regeneration of new myelin, a process called remyelination, occurs at the early stages of MS^2^. However, the body’s ability to remyelinate declines as the disease progresses or with increased age^2,3^. Although therapeutic strategies such as blocking the infiltration of immune cells into the CNS and limiting the function of peripheral immune cells are able to reduce the development of new lesions^4^, these drugs have limitations in targeting chronic active lesions inside the CNS which are associated with persistent inflammation, remyelination failure, and severe disability^5,6^.

Several studies have found that myeloid cells in the CNS, including microglia and macrophages, are the major immune cells that drive chronic inflammation, contributing to remyelination failure and disease progression in MS^5,7–10^. In early stages of MS when remyelination is more efficient, inflammatory microglia/macrophages have been proposed to transition to a pro-regenerative state, to promote oligodendrocyte differentiation and remyelination^11–15^. The pro-regenerative phenotype of microglia/macrophages in demyelinated lesions is critical for remyelination success. However, the mechanisms regulating the transition of microglia/macrophages from an inflammatory state to a pro-regenerative state in remyelinating lesions remain to be defined.

We have previously shown that interleukin-4 induced 1 (IL4I1) is upregulated in mouse lesions during a transitional period when remyelination initiates^16^. IL4I1 is an L-amino acid oxidase that converts aromatic amino acids into alpha-keto acids (AKAs). However, how IL4I1 mechanistically impacts remyelination remains unclear. Here, we found that IL4I1 is mainly produced by myeloid cells in remyelinating lesions. The disruption of IL4I1 expression in myeloid cells inhibited inflammation resolution and remyelination. The concentrations of AKAs (the main enzymatic products of IL4I1 activity) are reduced in lesions of mice lacking IL4I1 expression, and in the serum of people with MS at the progressive stage when remyelination is impaired. Administration of AKAs in mice following demyelination promoted inflammation resolution, and increased oligodendrocyte maturation and remyelination. Furthermore, AKAs altered the metabolic profile of myeloid cells in demyelinated lesions and activated aryl-hydrocarbon receptor (AhR) signaling. Our study suggests that the synthesis of AKAs in demyelinated lesions is necessary for efficient remyelination, and that increasing AKA levels in inflammatory demyelinating diseases, such as MS, may improve CNS repair.

## Results

### Expression of IL4I1 in myeloid cells is necessary for efficient remyelination

We previously found that IL4I1, an L-amino acid oxidase, is upregulated in lysophosphatidylcholine (LPC) demyelinated spinal cord lesions during remyelination^16^. However, it remained undetermined which cell population(s) express IL4I1 in demyelinated lesions, and how IL4I1 enzymatic activity affects remyelination efficiency. To determine which cell population(s) contribute to the expression of IL4I1, we used *Il4i1*^*tm1b/+*^ mice to tag one allele of the *Il4i1* gene with the *LacZ* reporter (Supplementary Fig. 1a), and performed immunostaining analysis at 10 days post lesion (dpl), when IL4I1 expression was expected^16^. We found that 89.76% of cells in lesions labeled with beta-galactosidase (β-gal) staining were CD11b^+^ myeloid cells, 4.15% of β-gal^+^ cells were CD3^+^ T cells, and 6.58% of β-gal^+^ cells were Sox9^+^ astrocytes (Supplementary Fig. 1b, c). β-gal expression in myeloid cells was further confirmed by colocalization with Iba1^+^ cells (Supplementary Fig. 1d). These data suggest that myeloid cells in lesions are the major source of IL4I1 during remyelination.

**Fig. 1:**
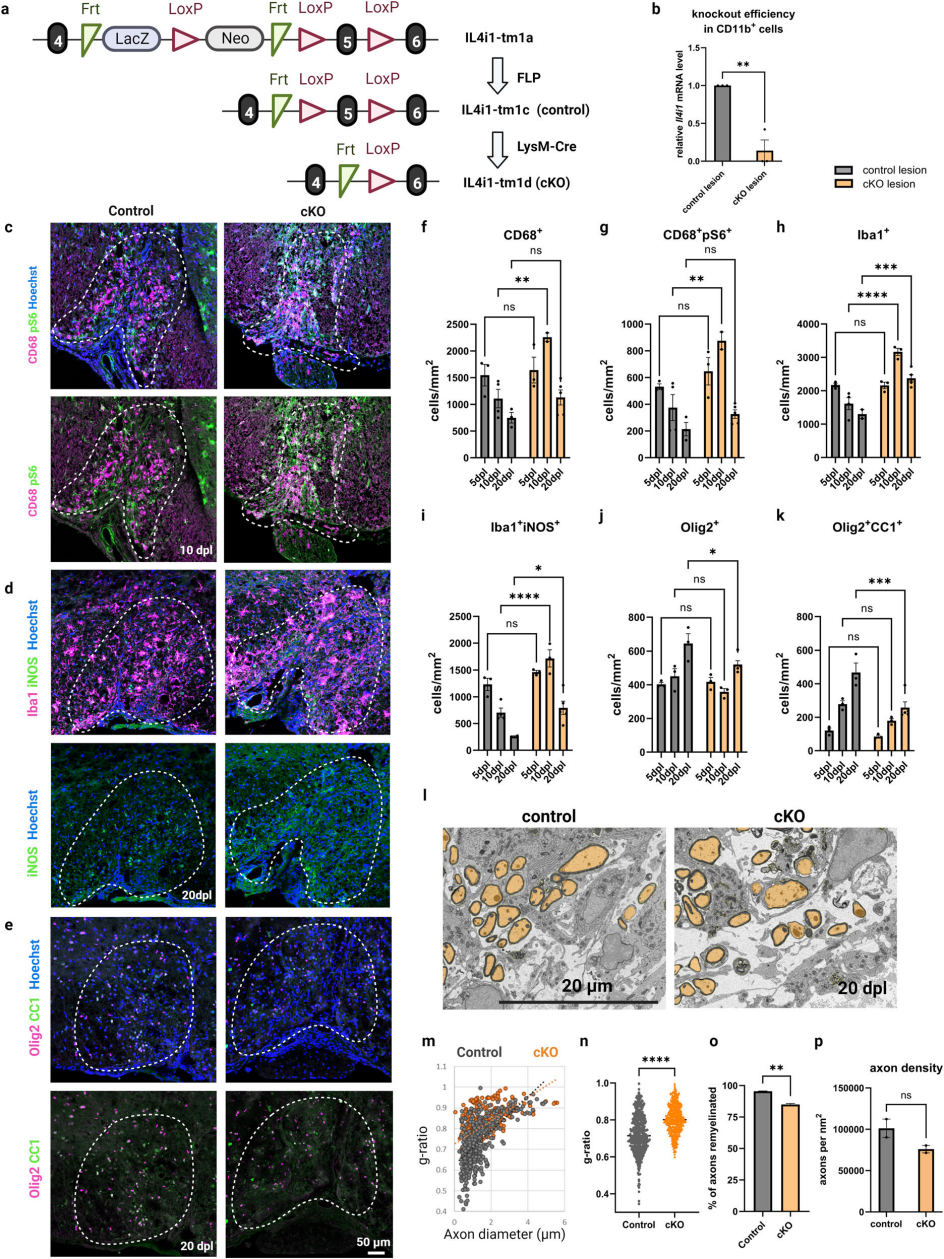
IL4I1 deletion in myeloid cells results in increased myeloid cell-driven inflammation and decreased remyelination. **a** Illustration of conditional knockout IL4I1 in myeloid cells and experimental controls. **b** RT-qPCR of *Il4i1* mRNA levels in MACS-isolated CD11b^+^ myeloid cells from the lesions of IL4I1-cKO mice and controls (*n* = 3, each *n* was obtained from 3–5 mice, unpaired *t* test). Representative images for immunostaining of mouse lesions for CD68^+^pS6^+^ myeloid cells at 10 dpl (**c**), Iba1^+^iNOS^+^ inflammatory myeloid cells at 20 dpl (**d**), and Olig2^+^CC1^+^ mature oligodendrocytes at 20 dpl (**e**). **f**–**k** Quantification of immunostaining cell counts in the lesions of IL4I1-cKO mice and littermate controls at 5, 10, 20 dpl (*n* = 3–5 mice, two-way ANOVA). **l** Representative images for SEM of spinal cord lesions of IL4I1-cKO mice and littermate controls at 20 dpl. **m**, **n** Quantification of g-ratio from SEM images (*n* = 2 mice for both groups, 195–296 axons from each *n*, unpaired *t* test). **o** Quantification of percentage of axons remyelinated (*n* = 2, unpaired *t* test). **p** Quantification of axonal density within the demyelinated lesions of controls and IL4I1-cKO mice. Bars represent the means with SEM. Each point represents an individual value. *P < 0.05, **P < 0.01, ***P < 0.001, ****P < 0.0001, ns = not significant.

We next determined whether IL4I1 expression in myeloid cells is involved in the remyelination process. To delete the *Il4i1* gene in myeloid cells, we crossed IL4I1-tm1c mice with a LysM-Cre line and generated *Il4i1*^*tm1c/tm1c*^*; Lyz2*^*Cre/+*^ conditional knockout mice (IL4I1-cKO) (Fig. 1a). Cre recombinase in LysM-Cre mice is known to be strongly expressed in microglia/infiltrating macrophages during CNS injury^17^ and is commonly used to induce gene deletion in myeloid cells^18,19^. We crossed the LysM-Cre line with a rosa reporter line and confirmed that 99.75% of Cre recombinase expression is in CD11b^+^ cells (Supplementary Fig. 2a, b, d–h). We observed that IL4I1-cKO mice are viable, fertile, and do not display any gross abnormalities. By RT-qPCR analysis on MACS-isolated CD11b^+^ myeloid cells from LPC-induced lesions at 10 dpl, when IL4I1 expression is high, we observed an 86% reduction in *Il4i1* mRNA levels in IL4I1-cKO mice (Fig. 1b). Since only 90.3% of CD11b^+^ cells are Lys2-Cre^+^ at 10dpl (Supplementary Fig. 2c), the incomplete removal of the *Il4i1* gene in CD11b^+^ cells suggest Cd11b^+^, Lys2^−^ cells might also produce IL4I1. To examine the impact of IL4I1-cKO on remyelination, we induced LPC demyelination, and performed immunostaining analysis of spinal cord lesions at 5 dpl (corresponding with high levels of inflammation and oligodendrocyte precursor cell (OPC) proliferation), 10 dpl (corresponding with inflammation resolution and oligodendrocyte differentiation), and 20 dpl (corresponding with remyelination)^16,20,21^.

We found that demyelinated lesions in both IL4I1-cKO and control mice exhibited similar size and demyelination pattern (Supplementary Fig. 3a, b). However, IL4I1-cKO mice displayed significantly more CD68^+^ myeloid cells at 10 dpl compared to *Il4i1*^*tm1c/tm1c*^*;Lyz2*^*+/+*^ littermate controls (Fig. 1c, f, *P* < 0.01). CD68 is an indicator of activated microglia/macrophages and has been observed at the hypercellular rim of chronic active lesions^5,22^. We found these cells also exhibited increased co-labeling with phospho-S6 ribosomal protein (pS6), a marker of mammalian target of rapamycin complex 1 (mTORC1) activity associated with activated microglial/macrophage responses^23,24^, at 10 dpl in the cKO lesion compared to the control lesions (Fig. 1c, g). Moreover, demyelinated lesions of IL4I1-cKO mice displayed significantly more Iba1^+^ myeloid cells with increased iNOS expression at 10 dpl (*P* < 0.0001) and 20 dpl (*P* < 0.05) compared to controls (Fig. 1d, h, i). These results suggest myeloid cell-driven inflammation is enhanced and unresolved in IL4I1-cKO mice.

To examine the effect of IL4I1-cKO on remyelination, we quantified the number of oligodendrocyte lineage cells in lesions and found a significant reduction of Olig2^+^ oligodendrocyte lineage cells and Olig2^+^CC1^+^ mature oligodendrocytes in IL4I1-cKO mice compared to control mice at 20 dpl (Fig. 1e, j, k, *P* < 0.05). Scanning electron microscopy (SEM) analysis of lesions at 20 dpl revealed thinner regenerated myelin membranes, as reflected by higher g-ratios, as well as a lower percentage of remyelinated axons in IL4I1-cKO mice compared to controls (Fig. 1l–o, and Supplementary Fig. 3c). To assess the extent of axonal loss in the IL4I1-cKO group, we analyzed axon density (the number of remyelinating axons normalized to lesion size) in IL4I1-cKO mice and littermate controls. While there was a trend of fewer axons in the IL4I1-cKO group, we did not observe a significant difference between the control and IL4I1-cKO mice (Fig. 1p). Together, these results suggest loss of IL4I1 function in myeloid cells results in failure to resolve microglia/macrophage-associated inflammation, and inefficient remyelination in lesions.

### IL4I1 generates alpha-keto acids (AKAs) in demyelinated lesions

**Fig. 2:**
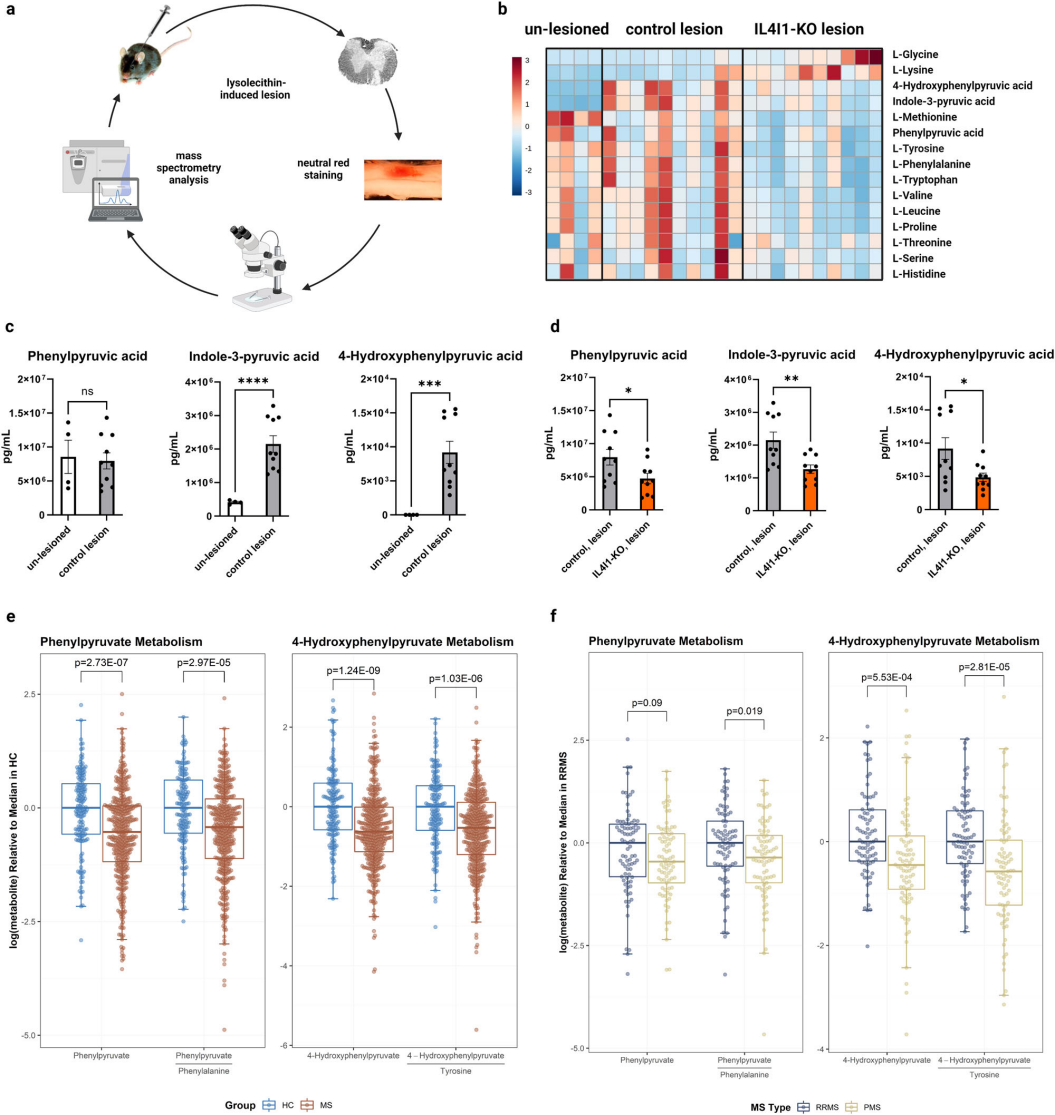
Metabolic profiling reveals decreased AKA levels in IL4I1 knockout mice and MS clinical samples. **a** Schematic of neutral red method isolating lesions for mass spectrometry. **b** Heatmap of amino acids and metabolites from un-lesioned tissues, WT control lesions, and IL4I1-KO lesions. **c** Quantification of phenylpyruvic acid (PPA), indole-3-pyruvic acid (IPA), and 4-hydroxyphenylpyruvic acid (HPPA) in un-lesioned tissues (*n* = 4 mice) and WT control lesions (*n* = 10 mice) at 10 dpl (Welch’s *t* test). **d** Quantification of PPA, IPA, and HPPA in WT control lesions (*n* = 10 mice) and IL4I1-KO lesions (*n* = 10 mice) at 10 dpl (Welch’s *t* test). **e** Box plots of PPA and HPPA metabolism in healthy controls (HC, *n* = 79) and people with multiple sclerosis (MS, *n* = 279). **f** Box plots of PPA and HPPA metabolism in age-matched people with relapsing-remitting MS (RRMS, *n* = 81) and progressive MS (PMS, *n* = 81). The upper limit of the box represents 75th percentile, the lower limit of the box represents 25th percentile and the central line represents median, while whiskers represent 1.5 times the interquartile range. Each point represents an individual value. *P < 0.05, **P < 0.01, ***P < 0.001, ns = not significant.

As an L-amino acid oxidase, IL4I1 has a preference for catalyzing the oxidation of aromatic amino acids, including phenylalanine, tryptophan, and tyrosine into phenylpyruvic acid (PPA), indole-3-pyruvic acid (IPA), and 4-hydroxyphenylpyruvic acid (HPPA), respectively^25,26^. To determine if IL4I1 expression affects the composition of amino acid metabolites in lesions during remyelination, mass spectrometry analysis was performed on demyelinated lesions dissected from *Il4i1*^*+/+*^ wild-type (WT) and *Il4i1*^*-/-*^ (IL4I1-KO) mice at 10 dpl, as well as from un-lesioned control tissues (Fig. 2a). IL4I1-KO mice were used here to exclude any metabolites produced by IL4I1. We found that demyelinated lesions in WT control animals exhibited similar overall amino acid levels as un-lesioned tissues at 10 dpl, (Fig. 2b and Supplementary Data 1), which is consistent with our previous finding that amino acid levels are reduced to baseline by this time point^20^. However, demyelinated lesions of IL4I1-KO mice were observed to display an overall shift in metabolism from those of WT mice (Fig. 2b). Analysis of the IL4I1-associated metabolites in WT mice revealed a significant increase in the levels of IPA and HPPA (*P* < 0.001), but not PPA, in control lesions compared to un-lesioned tissues, supporting the presence of IL4I1 activity in lesions at 10 dpl (Fig. 2c). Moreover, we found significant reductions of PPA, IPA, and HPPA in the lesions of IL4I1-KO mice compared to the lesions of WT control mice (Fig. 2d, *P* < 0.05). No difference in the levels of all three AKAs in non-lesioned tissues was observed between IL4I1-KO and WT mice (Supplementary Data 1). These results show that without IL4I1, there are lower concentrations of aromatic amino acid oxidative products (PPA, IPA, and HPPA) in demyelinated lesions, indicating the generation of these metabolites is dependent on the presence of IL4I1.

### Reduced AKA levels correlate with disease progression in MS

Recent studies have found that people with MS exhibit metabolic alterations in their blood compared to age- and sex-matched healthy controls (HCs)^27–29^. To determine if IL4I1-associated AKA levels were also altered in people with MS, we compared the serum levels of PPA and HPPA, as well as the ratios of PPA or HPPA to the aromatic amino acids from which they are derived, between people with MS and HCs. The demographics and clinical characteristics of the subjects and samples included across various analyses are shown in Table 1. We found that PPA (adjusted difference: −0.49 [95% CI: −0.68 to −0.3], *p* = 2.73E-07), PPA/phenylalanine ratio (adjusted difference: −0.41 [95% CI: −0.61 to −0.22], *p* = 2.97E-05), HPPA (adjusted difference: −0.61 [95% CI: −0.8 to −0.41], *p* = 1.24E-09) and HPPA/tyrosine ratio (adjusted difference: −0.5 [95% CI: −0.7 to −0.3], *p* = 1.03E-06) were all significantly decreased in people with MS compared to HCs (Fig. 2e). Next, to determine whether these altered metabolites are related to disease severity, we compared AKAs and AKA/parent-amino acid ratios between age-matched people with relapsing-remitting MS (RRMS) and progressive MS (PMS). We found the ratio of PPA/phenylalanine was significantly higher in people with RRMS (adjusted difference=0.38 [95% CI: 0.06–0.71], *p* = 0.02), as were both HPPA (adjusted difference=0.54 [95% CI: 0.24–0.84], *p* = 5.53E-04), and HPPA/tyrosine ratio (adjusted difference=0.63 [95% CI: 0.34–0.93], *p* = 2.81E-05) compared to those with PMS (Fig. 2f).

**Table 1:** Demographics and clinical characteristics.

Characteristic	All			Age-matched cohort			MRI
	HC (*N* = 79)	MS (*N* = 279)	*P* value	RRMS (*N* = 81)	PMS (*N* = 81)	*P* value	MS Cohort (*N* = 105)
Samples, *n*	156	408		81	81		105
Age, years, mean (SD)	42.0 (13.5)	44.4 (12.2)	0.17^a^	50.2 (8.9)	52.8 (9.9)	0.09^a^	43.7 (13.0)
Female, *n* (%)	50 (63%)	205 (73%)	0.08^b^	65 (80%)	53 (65%)	**0.03**^**b**^	78 (74)
Race, *n* (%)		**0.01**^**b**^		0.21^b^	-		
White	59 (75%)	237 (85%)		73 (90%)	66 (81%)		90 (86)
African American	10 (13%)	32 (11%)		7 (8.6%)	11 (14%)		10 (9.5)
Other	10 (13%)	10 (3.6%)		1 (1.2%)	4 (4.9%)		5 (4.8)
PMS, *n* (%)	-	94 (34%)					26 (25)
Time from blood draw, months, median (IQR)	-	-		-	-		6.0 (1.9–8.3)

^a^*p* values derived from two-sample t test.

^b^*p* values derived from chi-squared test.

Statistically significant values (<0.05) are bolded.

*HC* healthy controls, *IQR* inter-quantile range, *MS* multiple sclerosis, *PMS* progressive multiple sclerosis, *RRMS* relapsing-remitting multiple sclerosis, *SD* standard deviation.

In a subset of participants with available MRI data, we found that higher levels of PPA were significantly associated with higher cerebral white matter (WM) fraction (per 1 standard deviation (SD) increase in PPA: 0.005 [95% CI: 4e-04 to 0.009], *p* = 0.03) and with lower T2 lesion volume fraction (per 1 SD increase in PPA: −0.002 [95% CI: −0.003 to −4e−04], *p* = 0.01) (Table 2). A trend towards higher whole brain volume (WBV) was also observed (per 1 SD increase in PPA: 0.007 [95% CI: −1e−04 to 0.014], *p* = 0.053) (Table 2). Higher PPA/phenylalanine ratio was also associated with lower T2 lesion volume fraction (per 1 SD increase in the ratio: −0.002 [95% CI: −0.003 to −1e-04], *p* = 0.02) (Table 2). Moreover, increased levels of HPPA demonstrated a trend with decreased T2 lesion volume fraction (per 1 SD increase in HPPA: −0.001 [95% CI: −0.003 to 1e-04], *p* = 0.06), and similar trends were observed for other brain volume measures with HPPA but were not found to be statistically significant (Table 2).

**Table 2:** AKA association with MRI volumetrics.

Outcome^a^	PPA		PPA/phenylalanine		HPPA		HPPA/tyrosine	
	Beta (95% CI)	*p* value^b^	Beta (95% CI)	*p* value^b^	Beta (95% CI)	*p* value^b^	Beta (95% CI)	*p* value^b^
Cortical GM	0.003 (−2e−04 to 0.006)	0.06	0.003 (−3e-04 to 0.006)	0.07	0.003 (−7e−04 to 0.006)	0.12	0.003 (−8e-04 to 0.006)	0.13
Cerebral WM	0.005 (4e−04 to 0.009)	**0.03**	0.003 (−9e-04 to 0.008)	0.12	0.003 (−0.002 to 0.007)	0.24	0.003 (−0.001 to 0.007)	0.14
Subcortical GM	3e-04 (−2e−04 to 7e-04)	0.29	2e-04 (−2e-04 to 7e-04)	0.31	2e-04 (−2e−04 to 7e-04)	0.31	2e-04 (−2e-04 to 7e-04)	0.32
WBV	0.007 (−1e−04 to 0.014)	0.053	0.006 (−0.002 to 0.013)	0.13	0.006 (−0.001 to 0.013)	0.11	0.006 (−0.001 to 0.013)	0.1
T2 lesion	−0.002 (−0.003 to −4e−04)	**0.01**	−0.002 (−0.003 to -3e-04)	**0.02**	−0.001 (−0.003 to 1e-04)	0.06	−0.0011 (−0.003 to 3e-04)	0.13

Statistically significant values (<0.05) are bolded.

*GM* gray matter, *HPPA* 4-hydroxyphenylpyruvate, *PPA* phenylpyruvate, *WBV* whole brain volume, *WM* white matter.

^a^All MRI volumes are expressed as ratio of intracranial volume.

^b^*p* values derived from linear regression models adjusted for sex, age, and MS type.

Together, these results suggest that people with MS, especially at the progressive stage, display reductions of AKAs similarly to that observed in lesions of IL4I1-KO mice. A possible explanation for the lower levels of AKAs observed in PMS may be that the activity of IL4I1-related pathways is reduced or impaired during the later stages of MS. Our observations from MRI indicate a correlation between AKA concentrations and MRI parameters commonly used for assessing MS severity, suggesting that altered AKA metabolites may have an impact on the remyelination process and could play a role in the progression of MS.

### AKAs downregulate inflammatory myeloid cell activity to favor oligodendrocyte maturation in cell cultures

**Fig. 3:**
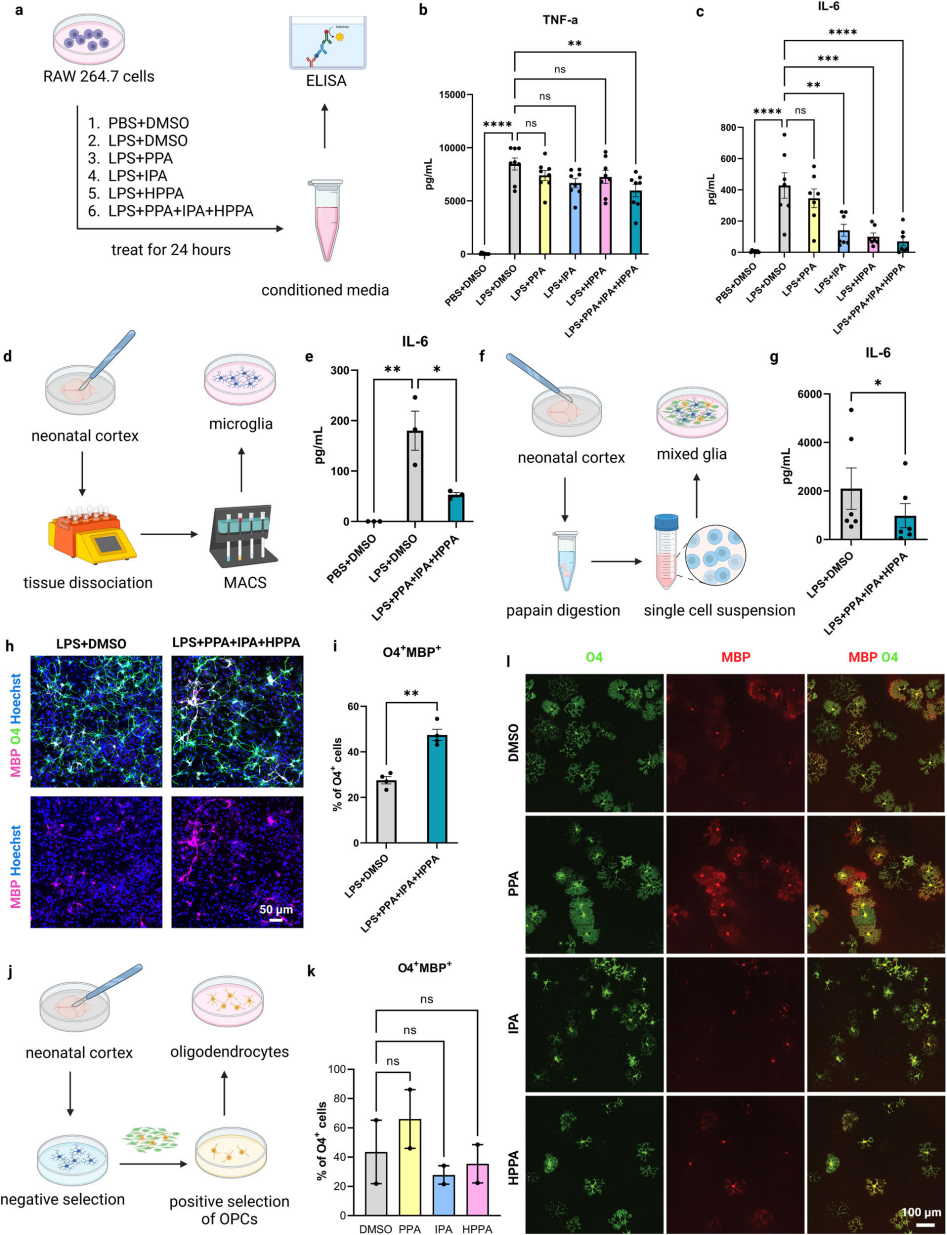
AKA treatment modulates myeloid cell inflammatory activity to favor oligodendrocyte maturation in vitro. **a** Schematic of experimental design for AKA treatment on RAW 264.7 cells. **b**, **c** ELISA analysis of TNF-α and IL-6 on conditioned media from un-stimulated or LPS-stimulated RAW 264.7 cells treated with PPA, IPA, or HPPA, or combination of the 3 AKAs (*n* = 7–8 independent replicates, Tukey’s multiple comparisons test). **d** Schematic of experimental design for AKA treatment on microglia. **e** ELISA result of IL-6 on conditioned media from un-stimulated or LPS-stimulated microglia with AKA treatment and DMSO control (*n* = 3 independent replicates, Tukey’s multiple comparisons test). **f** Schematic of experimental design for AKA treatment on mixed glial culture. **g** ELISA result of IL-6 on conditioned media from LPS-stimulated mixed glia treated with AKAs and DMSO controls (*n* = 6 independent replicates, paired *t* test). Representative images (**h**), and quantification (**i**) of MBP^+^O4^+^ mature oligodendrocytes in mixed glial culture treated with AKAs or DMSO in the presence of LPS (*n* = 4 independent replicates, paired *t* test). **j** Schematic of experimental design for AKA treatment on oligodendrocytes. Quantification (**k**), and representative images (**l**) of MBP^+^O4^+^ mature oligodendrocytes in oligodendrocyte culture treated with DMSO, PPA, IPA, or HPPA (*n* = 2 independent replicates, Kruskal–Wallis test). Bars represent the means with SEM. Each point represents an individual value. *P < 0.05, **P < 0.01, ***P < 0.001, ****P < 0.0001, ns = not significant.

To determine if IL4I1-associated AKAs affect macrophage activity under an inflammatory environment, we treated murine macrophage cell line RAW 264.7 with 250 μM PPA, IPA, or HPPA individually, or with a combination of all three AKAs under LPS stimulation. For control, RAW 264.7 cells under LPS stimulation were treated with volume-matched vehicle (DMSO). The conditioned media was then collected for enzyme-linked immunosorbent assay (ELISA) analysis of the inflammatory cytokines, tumor necrosis factor-alpha (TNF-α) and interleukin 6 (IL-6) (Fig. 3a). Because of the limitation of our method to detect the absolute AKA concentration in vivo, the doses of AKA treatments were based on a previous study^30^ and we confirmed that the treatments did not induce apoptosis by immunostaining analysis (Supplementary Fig. 4a, b) or change the pH of the cell culture media. We found that individual metabolites, when added to LPS-treated RAW 264.7 cells, did not significantly affect the level of TNF-α (Fig. 3b). However, the addition of IPA or HPPA alone was sufficient to lower the concentration of IL-6 (Fig. 3c, *P* < 0.01). Moreover, the addition of all three AKAs together significantly decreased the levels of TNF-α (Fig. 3b, *P* < 0.01) and IL-6 (Fig. 3c, *P* < 0.0001). To determine if IL4I1-associated AKAs affect microglial cell activity under an inflammatory environment, we next tested the effect of IL4I1-associated AKAs on MACS-isolated microglial cultures. We found that PPA + IPA + HPPA treatment also reduced IL-6 in LPS-stimulated microglia compared to DMSO controls (Fig. 3d, e). These results suggest that IL4I1-derived AKAs can directly decrease the inflammatory activity of LPS-stimulated myeloid cells.

Next, to determine whether the effect of IL4I1-associated AKAs on myeloid activity affects oligodendrocyte differentiation in vitro, we generated mixed glia cultures from the neonatal mouse cortex and treated the cultures with PPA + IPA + HPPA or DMSO (control) under LPS stimulation (Fig. 3f). We observed a reduction of IL-6 levels from the conditioned media (Fig. 3g, *P* < 0.05), and an increase in the percentage of O4^+^MBP^+^ mature oligodendrocytes in mixed glia treated with the AKA combination compared with DMSO (Fig. 3h, i, *P* < 0.01), suggesting IL4I1-associated AKAs promoted oligodendrocyte maturation in the presence of microglia under an inflammatory environment. To determine if IL4I1-associated AKAs directly affect oligodendrocyte maturation, PPA, IPA, HPPA, or DMSO were added separately to immunopanned primary OPC cultures for 24 h after 3 days in vitro (DIV) in differentiation media, followed by immunostaining analysis (Fig. 3j). We observed a potential increase in the percentage of O4^+^MBP^+^ mature oligodendrocytes with PPA treatment compared to DMSO control, but this observation did not reach statistical significance (Fig. 3k, l). Moreover, no difference in the percentage of O4^+^MBP^+^ oligodendrocytes was observed in IPA or HPPA-treated groups compared to the DMSO control (Fig. 3k, l). These findings suggest the effect of IL4I1-derived AKAs on oligodendrocyte maturation in mixed glia was likely mediated indirectly through modulating microglial inflammatory activity.

To determine if the observed effects of IL4I1-associated AKAs on microglia or oligodendrocytes align with the function of IL4I1, we repeated the in vitro experiments with recombinant mouse IL4I1 treatment. Compared to PBS controls, we found that IL4I1 treatment did not influence the oligodendrocyte population in OPC cultures (Supplementary Fig. 4c, e–g). This data was consistent with our prior finding that IL4I1 does not directly affect oligodendrocyte lineage cell progression^16^. We also tested the effect of recombinant IL4I1 treatment on oligodendrocytes in LPS-treated mixed glial cultures and observed an increase in the number of Olig2^+^ oligodendrocytes and the percentage of CC1^+^Olig2^+^ mature oligodendrocytes following IL4I1 addition compared to control (Supplementary Fig. 4d, h–j). The similar effects observed between IL4I1 and IL4I1-associated AKAs on oligodendrocytes and mixed glial cultures suggest that PPA, IPA, and HPPA may serve as the downstream effectors of IL4I1 activity.

### Oral AKA administration regulates inflammation and promotes remyelination in mice

**Fig. 4:**
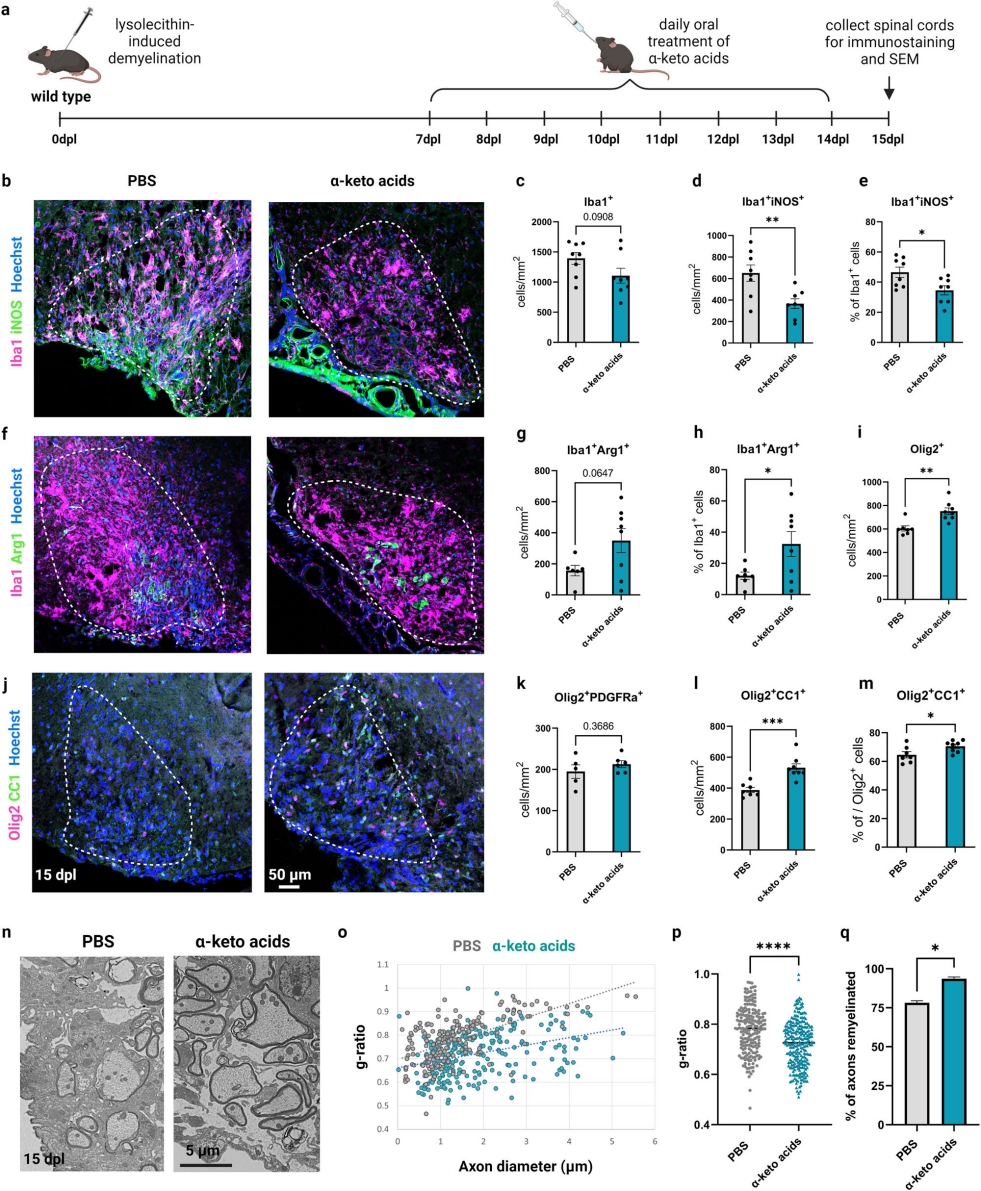
Therapeutic AKA administration resolves inflammation and enhances remyelination after LPC-induced demyelination in vivo. **a** Experimental design of AKA treatment on LPC-induced demyelinated mice. Representative images (**b**), and quantification (**c**–**e**) of Iba1^+^iNOS^+^ inflammatory myeloid cells in lesions from PBS or AKA-treated mice (*n* = 8 mice, unpaired t test). Representative images (**f**), and quantification (**g**–**h**) of Iba1^+^Arg1^+^ pro-regenerative myeloid cells in lesions from PBS or AKA-treated mice (*n* = 7–8 mice, unpaired t test). Representative images (**j**), and quantification (**i**, and **k**–**m**) of Olig2^+^ oligodendrocyte linage cells, Olig2^+^PDGFRα^+^ OPCs, and Olig2^+^CC1^+^ mature oligodendrocytes in lesions from PBS or AKA-treated mice (*n* = 5–8 mice, unpaired t test). **n** Representative images for scanning electron microscopy on lesions from PBS or AKA-treated mice at 15 dpl. **o**, **p** Quantification of g-ratios (*n* = 2 mice for both groups, 52–185 axons from each n, unpaired t test). **q** Quantification of percentage of axons remyelinated (*n* = 2, unpaired t test). Bars represent the means with SEM. Each point represents an individual value. *P < 0.05, **P < 0.01, ***P < 0.001, ****P < 0.0001.

To determine if the therapeutic administration of IL4I1-derived AKAs affects inflammation and remyelination in vivo, LPC-induced demyelination was performed on mice, followed by treatment with a combination of 200 mg/kg PPA, 200 mg/kg IPA, and 200 mg/kg HPPA through daily oral gavage from 7 to 14 dpl during the period of oligodendrocyte differentiation. Mice were then perfused at 15 dpl for immunostaining analysis (Fig. 4a). The dose of treatment was determined based on a previous study treating mice with chow containing 0.1% PPA, HPPA, or IPA^30^. We found that AKA-treated mice exhibited significantly decreased Iba1^+^iNOS^+^ inflammatory myeloid cells (Fig. 4b–e and Supplementary Fig. 5a, *P* < 0.05), and increased percentage of Iba1^+^Arg1^+^ pro-regenerative myeloid cells^14^ in lesions at 15 dpl compared to controls (Fig. 4f–h and Supplementary Fig. 5b), suggesting IL4I1-derived AKAs modulated myeloid cell activity in lesions towards an inflammation-resolved/pro-regenerative phenotype. Higher percentages of iNOS^+^ myeloid cells were found to be associated with less remyelination in MS lesions^10^. Since AKA treatment decreased iNOS^+^ myeloid cells, we also examined their effect on remyelination. We observed increased Olig2^+^ oligodendrocyte lineage cells (Fig. 4i, j), no difference in Olig2^+^PDGFRα^+^ OPCs (Fig. 4k), and increased Olig2^+^CC1^+^ mature oligodendrocytes with AKA treatment (Fig. 4j, l, m), suggesting that administration of IL4I1-associated AKAs increased oligodendrocyte differentiation in lesions. To examine the effect of IL4I1-associated AKAs on remyelination, we performed SEM analysis on lesions at 15 dpl and found that AKA treatment led to an increase in the thickness of regenerated myelin and the percentage of remyelinating axons compared to PBS-treated control mice (Fig. 4n–q). We further stratified the result based on the sexes of the mice, and found female mice responded better to AKA treatment. Although male mice showed similar trends of decreased myeloid cells and increased oligodendrocytes, the differences between AKAs and PBS-treated groups were only significant in female mice (Supplementary Fig. 6).

**Fig. 5:**
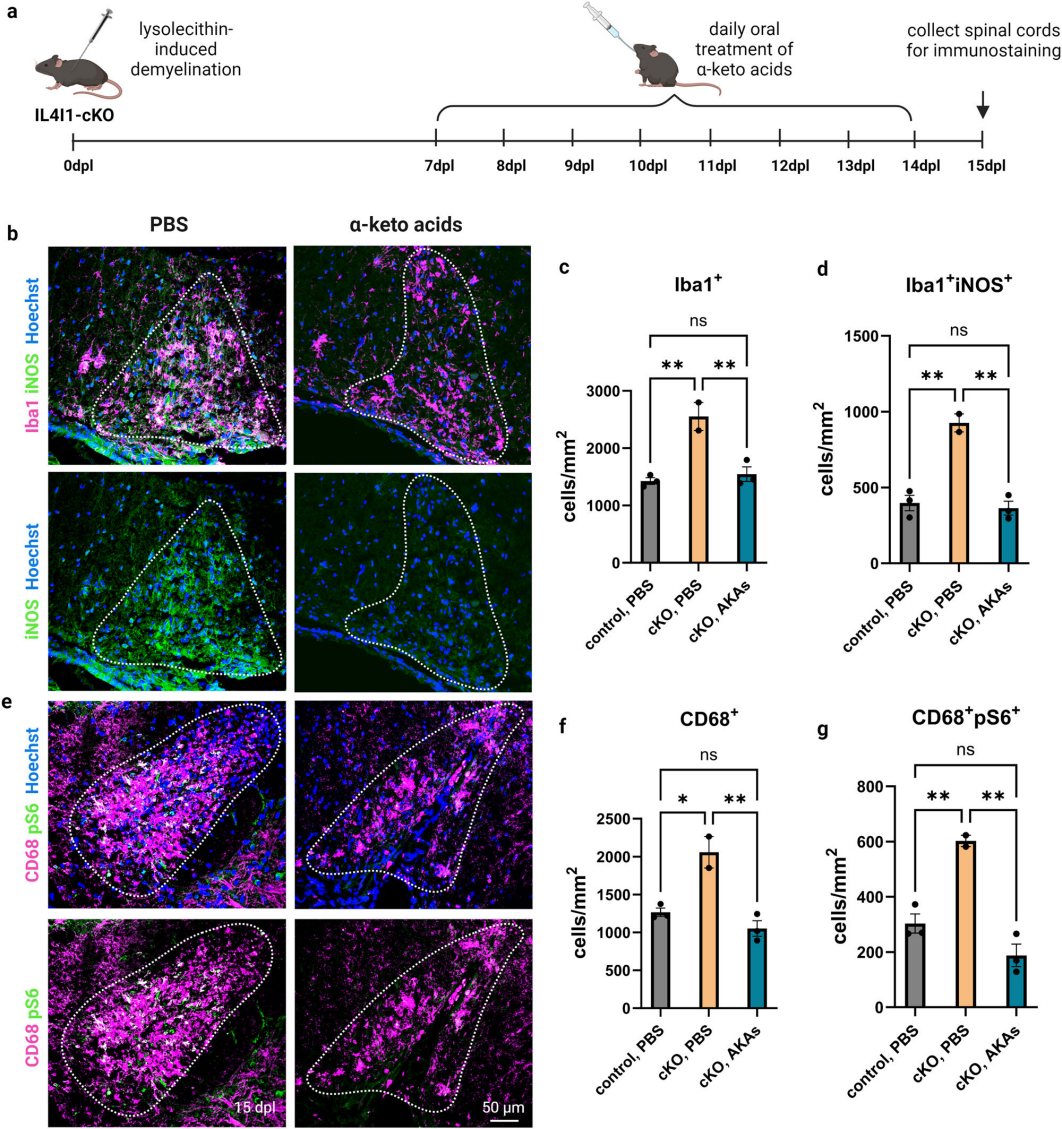
AKA administration of IL4I1-cKO mice. **a** Experimental design of AKA treatment of LPC-induced demyelinated IL4I1-cKO mice. **b–d** Representative images, and quantification of Iba1^+^ and Iba1^+^iNOS^+^ cells in lesions from PBS or AKAs-treated IL4I1-cKO mice and PBS-treated control mice (*n* = 2–3 mice, one-way ANOVA). **e–g** Representative images, and quantification of CD68^+^ and CD68^+^pS6^+^ cells in lesions from PBS or AKAs-treated IL4I1-cKO mice and PBS-treated control mice (*n* = 2–3 mice, one-way ANOVA). Bars represent the means with SEM. Each point represents an individual value. *P < 0.05, **P < 0.01, ns = not significant.

To determine if mice without IL4I1 respond to AKA treatment, we administered a mixture of 200 mg/kg PPA, 200 mg/kg IPA, and 200 mg/kg HPPA or the same volume of PBS control by daily oral gavage from 7 to 14 dpl to *Il4i1*^*tm1c/tm1c*^*;Lyz2*^*Cre/+*^ conditional knockout mice (IL4I1-cKO) and *Il4i1*^*tm1c/tm1c*^*;Lyz2*^*+/+*^ littermate controls after demyelination, and collected the tissues at 15 dpl for immunostaining analysis (Fig. 5a). Mice from both sexes were used in this study. Similar to the previous observation where an increase in inflammation was observed in the lesions of IL4I1-cKO mice, we found the cKO mice in the PBS group exhibited higher density of Iba1^+^ myeloid cells, Iba1^+^iNOS^+^ inflammatory myeloid cells, CD68^+^ MS-associated myeloid cells, and CD68^+^pS6^+^ activated myeloid cells compared to control mice with PBS treatment. (Fig. 5b–g). Moreover, compared to IL4I1-cKO mice treated with PBS, AKA administration significantly reduced the number of Iba1^+^, Iba1^+^iNOS^+^, CD68^+^, and CD68^+^pS6^+^ myeloid cells within the lesions of IL4I1-cKO mice (Fig. 5b–g). Altogether, these results suggest that administration of IL4I1-derived AKAs after demyelination modulated myeloid cell activity and enhanced remyelination efficiency in lesions.

### AKAs alter myeloid cell metabolism in demyelinated lesions

**Fig. 6:**
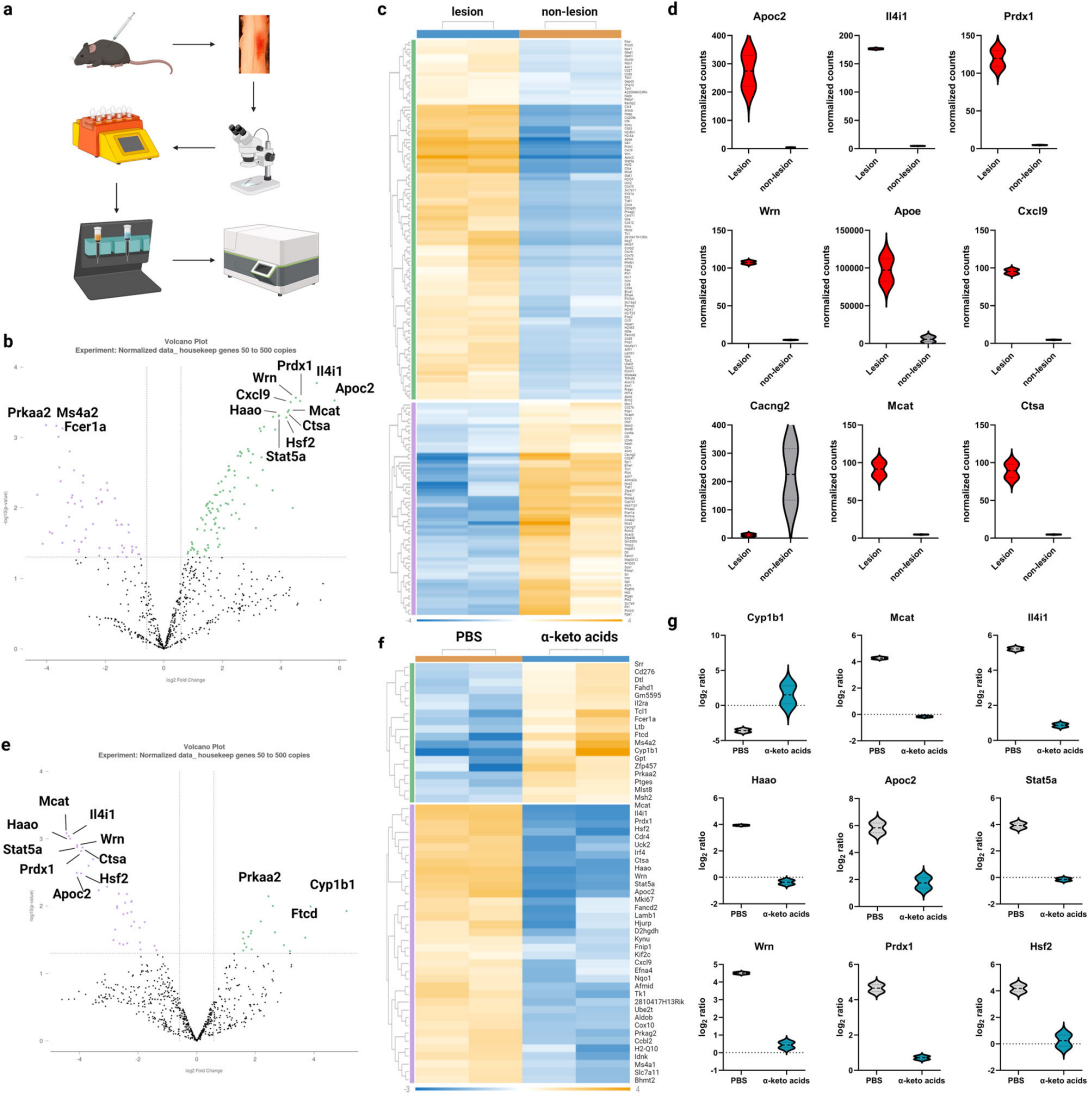
AKAs alter myeloid cell metabolism in lesions during remyelination. **a** Experimental design for myeloid cell isolation from mouse spinal cord lesions and Nanostring metabolic transcriptomic profiling. Volcano plot (**b**) and heatmap (**c**, *t* test, *p* < 0.05) for lesions and adjacent non-lesion tissues. **d** Violin plot for top 9 differentially expressed genes between lesion and adjacent non-lesion tissues (*n* = 2, each *n* was obtained from 3–5 mice, t test, *p* < 0.05). Volcano plot (**e**) and heatmap (**f**) for differential gene expression between lesions from PBS-treated and AKAs-treated mice (*t* test, *p* < 0.05). **g** Violin plot for top 9 differentially expressed genes between lesions from PBS-treated and AKAs-treated mice (*n* = 2, each n was obtained from 3–5 mice, *t* test, *p* < 0.05).

To determine the downstream targets of IL4I1-associated AKAs in modulating CNS inflammation, we isolated CD11b^+^ cells from focally demyelinated lesions and non-lesioned control tissues by magnetic-activated cell sorting (MACS) after LPC-induced demyelination in the mouse spinal cord white matter (Fig. 6a). Differential expression of genes associated with cellular metabolism were analyzed using the NanoString nCounter mouse metabolic pathway panel at 15 days post-lesion (dpl) during remyelination^20,31^. In PBS-treated mice, we found that 159 genes in myeloid cells were significantly differentially expressed in lesions compared to non-lesioned controls, including *Il4i1* (amino acid metabolism), *Apoc2*, *Apoe* (lipid metabolism), *Prdx1*, *Wrn* (reactive oxygen response), *Cxcl9* (cytokine/chemokine signaling), *Cacng2* (previously detected in bone marrow-derived macrophages and microglia with unknown function^32^), *Mcat* (fatty acid metabolism), *Ctsa* (lysosomal degradation), and *Hsf2* (heat-shock response) (Fig. 6b–d, *P* < 0.05, Supplementary Data 2). In particular, *Il4i1* was one of the most upregulated transcripts in myeloid cells from remyelinating lesions (Fig. 6d), thus confirming our previous data that IL4I1 expression increases during remyelination.

**Fig. 7:**
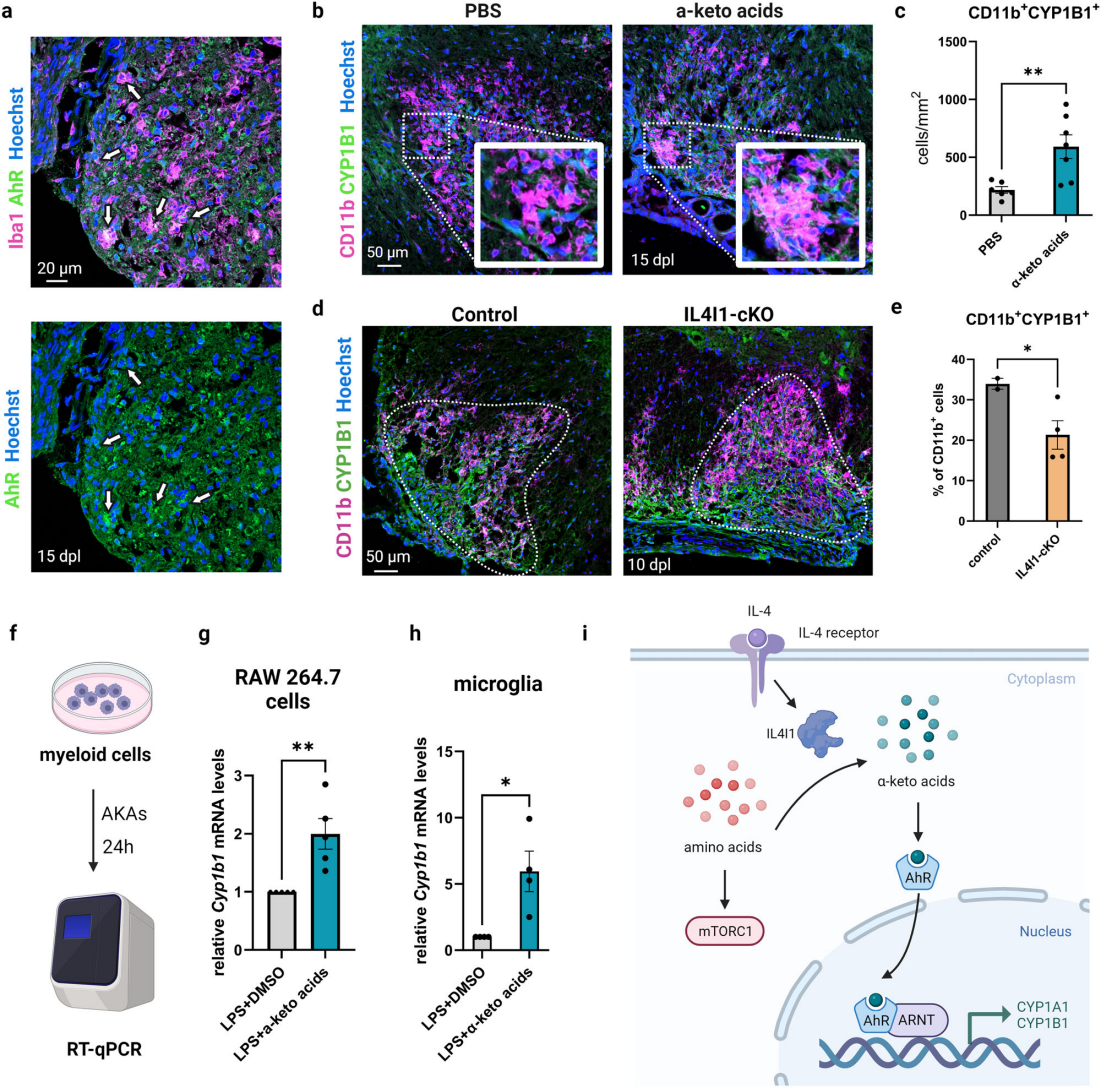
AKAs stimulate AhR signaling in myeloid cells. **a** Representative images of immunostaining for Iba1 (myeloid cell marker) and AhR (aryl hydrocarbon receptor) in lesions from PBS-treated control mice at 15 dpl. Arrows pointing at Iba1^+^AhR^+^ cells. The scale bar represents 20 μm. **b** Representative images of immunostaining for CD11b (myeloid cell marker) and CYP1B1 (AhR activity indicator) in the lesions from PBS-treated and AKA-treated mice. The scale bar represents 50 μm. **c** Quantification of CD11b^+^CYP1B1^+^ cells in lesions from PBS-treated and AKA-treated mice (*n* = 6–7 mice, unpaired *t* test). Bars represent the means with SEM. Each point represents an individual value. **d** Representative image of CD11b and CYP1B1 immunostaining on control and IL4I1-cKO lesions at 10 dpl. The scale bar represents 50 μm. **e** Quantification of the percentage of CD11b^+^CYP1B1^+^ cells over all CD11b^+^ cells in the lesions (n = 2–4 mice, unpaired t test). **f** Schematic of AKA treatment on myeloid cells for RT-qPCR. **g**–**h** qRT-PCR of *Cyp1b1* mRNA expression normalized to housekeeping genes (*Rpl13a* and *Ppia*) on control or AKA-treated RAW 264.7 cells (*n* = 5) and microglia (*n* = 4 independent replicates, unpaired *t* test). **i** A model of IL4-IL4I1-AKAs-AhR axis in myeloid cells during remyelination. Bars represent the means with SEM. Each point represents an individual experiment. *P < 0.05, **P < 0.01.

To examine the effect of AKA treatment on myeloid cells, the expression level of each gene in lesions was first normalized to the non-lesioned controls from the same sample, and then further compared between the PBS and AKAs groups. We found a metabolic shift, including 54 genes, that were significantly altered after AKA treatment (Fig. 6e–g, *P* < 0.05, Supplementary Data 3). The top differentially expressed genes were involved in tryptophan metabolism (*Cyp1b1*, *Haao*), fatty acid metabolism (*Mcat*), amino acid metabolism (*Il4i1*), lipid metabolism (*Apoc2*), cytokine/chemokine signaling (*Stat5a*), reactive oxygen species response (*Wrn*, *Prdx1*), and heat-shock response (*Hsf2*) (Fig. 6g). These results indicate that IL4I1-associated AKAs reprogrammed the metabolic state of myeloid cells in lesions. Among all the transcripts detected by NanoString, we found *Cyp1b1* was the most significantly upregulated gene in myeloid cells after AKA treatment (Fig. 6g, P < 0.05). *Cyp1b1* (Cytochrome P450 family 1 subfamily B member 1) is an enzyme that functions downstream of the aryl hydrocarbon receptor (AhR) and has been used as an indicator for AhR activation^33^. AhR is a ligand-activated transcription factor that senses ligands from the environment and regulates immune responses^34^. We performed immunostaining analysis of demyelinated lesions at 15 dpl and confirmed that AhR is expressed in Iba1^+^ myeloid cells (Fig. 7a). Moreover, we confirmed that AKA treatment increased CYP1B1^+^CD11b^+^ myeloid cells in lesions compared to the PBS-treated controls via immunostaining (Fig. 7b, c). These results suggest that IL4I1-derived AKA administration stimulates AhR signaling in myeloid cells during remyelination.

To determine if loss of IL4I1 function affects the activity of AhR signaling in myeloid cells, we examined the expression of CYP1B1 in CD11b^+^ cells within the lesions of IL4I1-cKO compared to littermate controls after LPC-induced demyelination. We found that IL4I1-cKO mice displayed a decreased percentage of CD11b^+^CYP1B1^+^ cells out of total CD11b^+^ cells in the lesions (Fig. 7d, e, *P* < 0.05), indicating myeloid cells exhibit reduced AhR signaling in the absence of IL4I1. To determine if IL4I1-derived AKAs directly affect AhR activity in myeloid cells, we treated macrophage and microglial cultures with PPA + IPA + HPPA or DMSO control under LPS stimulation in vitro, followed by RT-qPCR respectively (Fig. 7f). We found that AKA treatment significantly increased the mRNA level of *Cyp1b1* in both RAW 264.7 macrophage (Fig. 7g, *P* < 0.01) and primary microglia cultures (Fig. 7h, *P* < 0.05). To determine if the activation of AhR pathway is necessary for the effect of AKAs on oligodendrocyte maturation, we examined the effect of an AhR inhibitor (CH-223191) on oligodendrocytes in mixed glia cultures under LPS stimulation. We found that the effect of AKAs on oligodendrocyte maturation in mixed glial culture was significantly impaired when AhR activity was inhibited by the inhibitor CH-223191 (10 μM) (Supplementary Fig. 7). This observation suggests that the AhR pathway is an essential downstream target of IL4I1-derived AKAs. Taken together, our results indicate the IL4I1-AKAs-AhR pathway plays an important role in the resolution of inflammation, which in turn is critical for oligodendrocyte differentiation and maturation during the remyelination process (Fig. 7i).

## Discussion

A major obstacle to remyelination in MS is the lack of therapeutics targeting chronic CNS inflammation in demyelinated lesions^35^. Understanding the regulation of myeloid cell activity during spontaneous remyelination may lead to the development of new drug targets to improve myelin repair in diseases like MS. Metabolic changes, or metabolic reprogramming, have been suggested to regulate the activation states of myeloid cells^36–38^. For example, inflammatory myeloid cells rely on aerobic glycolysis to maintain cytotoxicity, while oxidative phosphorylation and fatty acid oxidation shape myeloid cells towards an immunosuppressive phenotype^39^. As such, the intrinsic metabolic state of myeloid cells in demyelinated lesions may be associated with remyelination efficiency.

Through profiling of metabolic genes expressed in myeloid cells from LPC-induced demyelinated lesions in mice, we found genes associated with amino acid metabolism, such as *Il4i1* (interleukin 4 induced 1), *Haao* (3-hydroxyanthranilate 3,4-dioxygenase), *Kynu* (kynureninase), and Nos2 (nitric oxide synthase 2, inducible) were significantly differentially expressed in lesions compared to adjacent non-lesion control tissues (Supplementary Data 2). Our previous studies suggest there is an accumulation of amino acids in the lesion microenvironment after demyelination, followed by clearance upon remyelination^20^. It is possible that the metabolism of amino acids is an essential component of remyelination. Among all 748 genes in the Nanostring metabolic panel, *Il4i1* was the most significantly upregulated gene (log_2_ Fold Change=5.22, *p* = 0.0002). The expression of IL4I1 is not only seen in mice but also in individuals with MS, in which the increase in the expression of the *Il4i1* gene could be detected in both normal appearing white matter (NAWM, log Fold change= 2.39, *p* = 0.00032) and demyelinated white matter lesions (WMLs, log Fold change= 2.88, *p* = 0.00010) of MS donors compared to white matter of control donors (CWM)^40^.

While IL4I1-related changes were our main focus, other genes/pathways were illuminated with the Nanostring metabolic panel that may be crucial for remyelination. We found genes responsible for lipid metabolism and fatty acid synthesis, such as *Apoc2* (apolipoprotein C2), *Mcat* (malonyl CoA:ACP acyltransferase), *Apoe* (apolipoprotein E), *Pik3r3* (phosphoinositide-3-kinase regulatory subunit 3), and *Apob* (apolipoprotein B), were also significantly differentially expressed in myeloid cells during remyelination (Supplementary Data 2). Lipid mediators have been reported to modulate the inflammatory reactions of microglia/macrophages within demyelinating lesions^41^. Apolipoprotein such as ApoE has also been reported to modulate myelin debris clearance and microglial immunometabolism^42,43^. Further studies exploring other genes/pathways/molecules in the lesions discovered by our Nanostring analysis may provide more insight into the modulation of myeloid cell activity during remyelination.

As a downstream product of interleukin-4 (IL-4) signaling, IL4I1 drives immunosuppressive effects usually seen in cancers^44,45^. In our animal model of remyelination, we found that the expression of IL4I1 by myeloid cells is important for remyelination, as conditional knockout of IL4I1 in myeloid cells inhibited remyelination. By mass spectrometry analysis, we found that IL4I1-KO mice exhibit lower levels of AKAs (PPA, IPA, and HPPA). Moreover, the levels of IL4I1-derived AKAs and the ratios of AKA to corresponding amino acid are reduced in people with MS, and this decline correlates with more severe disease. The reduction of these metabolites in people with MS may contribute to a similar microenvironment to what we observed in the lesions of IL4I1-KO mice, where inflammation remains unresolved and remyelination is impaired. However, we also unexpectedly observed an overall decrease of amino acid levels in the lesions of IL4I1-KO mice including L-valine, L-leucine, L-tyrosine, L-proline, L-tryptophan, L-phenylalanine, L-lysine, and L-methionine. The only amino acid that was observed to increase in IL4I1-KO lesions compared to IL4I1-WT control lesions was L-glycine. L-glycine has been found to suppress the activation of inflammatory macrophages^46^. The increased glycine levels might serve as a compensatory mechanism to resolve inflammation with the loss of IL4I1. The significant reduction in the concentrations of methionine that we observed in IL4I1-KO lesions has also been reported in plasma from people with RRMS compared to healthy controls^47^. Reductions in tryptophan and phenylalanine in serum were also found associated with higher clinical disability in MS^28^. One explanation for the reduction of these amino acids in MS serum and in IL4I1-KO lesions is that there might be a compensatory increase in the effect of other enzymes/pathways responsible for amino acid metabolism. For example, an analysis of amino acid metabolism in MS serum samples revealed alterations in the ratios of methionine to glycine (converted by methionine-glyoxylate transaminase) and leucine to N-acetyl-leucine (converted by leucine N-acetyltransferase) compared to the controls^28^. Further studies analyzing the products of other amino acid metabolism pathways or examining the activities of other enzymes involved in amino acid metabolism are needed to fully explain the differences we observed in amino acid concentrations in the IL4I1-KO demyelinated lesions.

To test the effect of IL4I1-derived alpha keto acids on inflammation, we stimulated myeloid cell cultures with LPS and treated them with PPA, IPA, HPPA, or the combination of the three. We found that treatment of myeloid cells with the combination of PPA, IPA, and HPPA together was effective in decreasing inflammatory activity induced by LPS. In particular, IPA or HPPA alone was sufficient to decrease the concentration of inflammatory cytokine IL-6, which is supported by a previous study showing IPA downregulated the expression of Th1 and proinflammatory cytokines in the colon^30^, suggesting that IPA and HPPA might be more effective in regulating inflammation. Moreover, both AKAs and IL4I1 treatment increased oligodendrocyte differentiation in LPS-stimulated mixed glial culture, suggesting that IL4I1 metabolically modulates the inflamed microenvironment by producing AKAs to promote oligodendrocyte differentiation in the presence of microglia. Additionally, we found none of the AKAs or IL4I1 was able to significantly influence oligodendrocyte lineage cell progression when added to pure OPC cultures without microglia, suggesting the effect of IL4I1-derived AKAs on oligodendrocyte maturation is indirect, only functioning in the presence of myeloid cells.

To confirm the observed in vitro results using an animal model of remyelination, we therapeutically treated LPC-demyelinated mice with AKAs, and found that AKA treatment led to inflammation resolution and enhanced remyelination in lesions. However, this effect was only statistically significant in female mice. It is possible that lesions from male and female mice respond differently to AKA treatment. We observed that myeloid density in female mice (1500/mm^2^) is slightly higher than in male mice (1250/mm^2^). It is possible that female mice with severe inflammation are more responsive to AKA treatment. In IL4I1-cKO mice, mice from both sexes exhibit high levels of inflammation (2500 myeloid cells/mm^2^) and displayed a significant reduction in the density of activated myeloid cells with AKA treatment. Since AKAs were administered through oral gavage, we could not rule out the possibility that the observed improved effect of AKAs on inflammation and remyelination might also be linked to their influence on peripheral immune cells or other cell type outside of the CNS.

We also explored the downstream targets of the IL4I1-AKAs axis with NanoString metabolic pathway analysis by comparing lesions from AKAs-treated mice and PBS-treated mice. Interestingly, many genes associated with metabolism (such as *Apoc2, Il4i1, Prdx1, Wrn, Mcat, Ctsa, Hsf2, Haao*) that were upregulated in the lesions compared to non-lesioned tissues, were decreased with AKA treatment, indicating that AKAs brought the microenvironment to baseline/homeostatic level similar to that in non-lesioned tissues. Curiously, AKA treatment also decreased *Il4i1* expression, which suggested a potential negative feedback mechanism of *Il4i1* expression through IL4I1-generated AKAs in lesions. In this study, we identified AhR signaling as a major target of IL4I1-derived AKAs on myeloid cells. The result is supported by previous publications suggesting AKAs modulate inflammation by acting as ligands for AhR signaling in experimental colitis and cancer^26,30^. Studies have found that mice lacking AhR expression during development exhibit increased inflammation and corresponding alteration of myelin sheaths in optic nerves^48^. Further, experimental autoimmune encephalomyelitis (EAE) induction in mice lacking AhR expression resulted in increased inflammatory demyelination and clinical disability^49^. These studies suggest that the AhR pathway is a critical regulator of inflammation, and that deficient AhR signaling enhances inflammatory demyelination. Indeed, lower AhR agonistic activity and decreased levels of AhR-activating molecules have been observed in MS serum compared to HCs^50^. It is worth noting that AhR activation can drive both inflammatory and anti-inflammatory effects depending on the ligands^34,51^. From our study, we found that the AhR pathway is activated with AKA treatment, and that mice lacking IL4I1 display lower AhR activity. Moreover, we found that IL4I1-associated AKA treatment decreased the levels of inflammatory cytokines TNF-α and IL-6 in vitro and decreased the number of inflammatory Iba1^+^iNOS^+^ myeloid cells in vivo. These findings suggest IL4I1 targets AhR through the generation of AKAs, and that AKAs mediate anti-inflammatory responses in myeloid cells in lesions during remyelination. The activation of AhR signaling by AKAs might be an important regulator of myeloid cell activity in lesions during remyelination, but further studies with genetic or pharmacological approaches are needed to confirm the function of AhR in remyelination.

Spontaneous remyelination, from an immune perspective, requires anti-inflammatory immune regulation, efficient phagocytosis of myelin debris, and the replacement of infiltrating peripheral macrophages by resident microglia^52^, Moreover, pro-inflammatory microglia in demyelinated lesions have been shown to undergo necroptosis before the lesion is repopulated by pro-regenerative microglia for remyelination^15^. The loss of IL4I1 in myeloid cells could possibly affect one or several of the above mechanisms. Future studies delineating the effects of IL4I1 on separate myeloid populations are needed to fully address our understanding of myeloid cell dynamics during remyelination. In conclusion, our data suggests IL4I1-associated AKAs are generated in demyelinated lesions during remyelination and regulate myeloid cell metabolism by activating AhR signaling to facilitate myelin repair. Therapeutic approaches using IL4I1-derived AKAs may serve as potential treatments for regulating myeloid cell-driven inflammation, and improving CNS remyelination in inflammatory demyelinating diseases such as MS.

## Methods

### Animals

All experiments were performed in accordance with approved Institutional Animal Care and Use Committee (IACUC) protocols of Georgetown University. C57BL/6 mice (RRID: IMSR_JAX:000664), ACTBFLPe mice (RRID: IMSR_JAX:005703), and LysMcre knock-in/knock-out mice (RRID: IMSR_JAX:004781) were purchased from the Jackson Laboratory. B6.Cg-Gt(ROSA)26Sortm14 (RRID: IMSR_JAX:007914) reporter line was a gift obtained from the Coate lab in the Department of Biology at Georgetown University. IL4I1-KO (*Il4i1*^*-/-*^) mice (RRID: MMRRC_011726-UCD) were purchased from Mutant Mouse Resource & Research Centers (MMRRC). IL4I1-tm1a (MGI: 4432453) and IL4I1-tm1b (MGI: 6120692) mice were purchased from European Mouse Mutant Archive (EMMA). Timed pregnant Crl:CD(SD) rats (RRID: RGD_734476) were purchased from Charles River. For experiments, IL4I1-KO mice and IL4I1-tm1b mice were outbred with C57BL/6 mice and then inbred with heterozygous mice, respectively, to generate mutant and wild-type littermate controls. IL4I1-tm1a mice were bred with ACTBFLPe mice to generate IL4I1-tm1c, and then with LysMcre mice to generate IL4I1-tm1d. Then *Il4i1*^*tm1c/tm1c*^*; Lyz2*^*Cre/+*^ (IL4I1-tm1d) were backcrossed with *Il4i1*^*tm1c/tm1c*^*; Lyz2*^*+/+*^ (IL4I1-tm1c) to generate *Il4i1*^*tm1c/tm1c*^*; Lyz2*^*Cre/+*^ mice (IL4I1-tm1d, cKO) and *Il4i1*^*tm1c/tm1c*^*; Lyz2*^*+/+*^ littermate controls (IL4I1-tm1c, control). Mice of both sexes were used in this study. All above animals were housed at the Georgetown University Division of Comparative Medicine following IACUC protocol and maintained on a 12-h-light/dark cycle with food and water *ad libitum*. Sprague-Dawley rats used to prepare OPC cultures for AKA treatment were shipped as moms and P5 pups from Charles River and housed at the vivarium in the John Edward Porter Neuroscience Research Center (PNRC) at NINDS.

### Human cohort

Serum samples were collected from Johns Hopkins MS Center research participants with MS (*n* = 279) and HCs (*n* = 79). Samples were drawn at different time points throughout the longitudinal follow-up of these people and were not pre- or post-treatment samples. Blood was processed within 3 h of the time of collection and serum aliquots were stored at −80 °C until metabolomics analyses. Written informed consent was obtained from the participants to participate in the study.

### Brain magnetic resonance imaging (MRI)

A fraction of the MS cohort (*N* = 105) had brain MRIs within a year of serum collection. Harmonization and segmentation of the MRI scans were performed, and segmentations were manually reviewed for segmentation errors/failures, as previously described^53^. Volumes of bilateral structures were calculated as the sum of the left and right volumes of the corresponding structure. All volumes were normalized to the median intracranial volume for each participant, to account for inter-person skull size variability. Fractions for cortical gray matter (GM), subcortical GM and WBV were calculated by summing the volume fractions of their respective components. We assessed the association between MRI volumetrics and individual metabolites and metabolite ratios in people with MS, utilizing linear regression models adjusted for sex, age, and MS type. All analyses were performed using R software, version 4.2.0 (https://www.r-project.org/). Statistical significance was defined as *p* < 0.05.

### Metabolomics analyses of human samples

Metabolomics analyses were performed at Metabolon Inc. (Durham, NC) as previously described^27^. Serum samples were collected and a methanol-based extraction was performed to generate the fractions that were then used for downstream analysis, as previously described^54^. Briefly, recovery standards were added prior to the first step in the extraction process for quality control purposes. To remove protein, dissociate small molecules bound to protein or trapped in the precipitated protein matrix, and to recover chemically diverse metabolites, proteins were precipitated with methanol under vigorous shaking for 2 min (Glen Mills Genogrinder 2000) followed by centrifugation. The resulting extract was divided into five fractions: one for analysis by ultra-high performance liquid chromatography tandem mass spectrometry (UPLC-MS/MS; positive ionization), one for analysis by UPLCMS/MS (negative ionization), one for the UPLC-MS/MS polar platform (negative ionization), one for analysis by gas chromatography–mass spectrometry (GC-MS), and one sample was reserved for backup.

Liquid chromatography with tandem mass spectrometry (LC-MS/MS) or gas chromatography with mass spectrometry (GS/MS) was then performed and the subsequent mass spectra were matched to a reference library for metabolite identification. Subsequently, the area under the curve was used for the quantification of each metabolite level. There were five different runs of metabolomic analyses in 564 total samples, including both MS and HC. Metabolites with ≥30% missing values across samples for each run were excluded from the analysis and the rest of the missing values were imputed using k-nearest neighbors (3 neighbors used for each imputation), resulting in 320 common metabolites. Metabolites were log-transformed, and we adjusted for batch effects using the ComBat algorithm^55^.

Analyses were focused on the available keto acids (PPA, HPPA) and their ratios to their parent amino acids (PPA/phenylalanine, HPPA/tyrosine). IPA was not identified in the assay that we used. The metabolite ratios of interest were calculated using the raw concentration of the metabolites after batch adjustment and then they were log-transformed for the analyses. For easier interpretation of the results, all metabolites and ratios analyzed were scaled, and divided by the standard deviation of each variable. We compared individual log-transformed metabolites and ratios of interest between HC and people with MS, utilizing generalized estimating equations, in order to account for multiple metabolomic profiles/samples contributed by some participants. Analyses were adjusted for gender, age, and race (white, African American, other). We then compared the same metabolites between age-matched people with RRMS and PMS (either primary or secondary PMS), using linear regression models adjusted for gender and race (only one sample per subject). All analyses were performed using the R software, version 4.2.0 (https://www.r-project.org/). Statistical significance was defined as *p* < 0.05.

### Lysolecithin-induced spinal cord focal demyelination mouse model

Mice at 8–12 weeks old from both sexes were used for the spinal cord focal demyelination model. In brief, 1 μL of 1% lysophosphatidylcholine (LPC) dissolved in sterile PBS was injected into the ventral white matter of the mouse spinal cord as previously described^56^. Analgesic Carprofen (5 mg/kg) was delivered subcutaneously once before surgery, and once 24 h after surgery. For post-procedural pain relief, 0.1 ml of 0.025% Bupivacaine was delivered once by intra-incisional infusion at the surgical site. Perfusion was performed under isoflurane-induced anesthesia at 5–20 days post lesion (dpl) to examine remyelination. Remyelination of IL4I1-KO mice was monitored at 5, 10, and 20 dpl. Remyelination of AKAs-treated mice was observed at 15 dpl following the last day of treatment.

### In vivo AKA treatment

AKAs were suspended in PBS at 200 mg PPA (Millipore Sigma) + 200 mg IPA (Santa Cruz) + 200 mg HPPA (Millipore Sigma) per kg body weight and provided to the mice by gavage. Focal demyelinated mice were treated with AKAs daily from 7 dpl to 14 dpl. The control group received the same volume (100 μL) of PBS via gavage. To prevent coprophagy, mice in AKA treatment and control groups were housed in separate cages from the beginning of treatments.

### Myeloid cells isolation from spinal cord lesions

To detect and isolate demyelinated lesions in the spinal cords, mice were intraperitoneally injected with 0.5 mL 1% neutral red in sterile PBS 2 h before PBS perfusion, as previously described^20^. 3–5 dissected spinal cord lesions at 15 dpl and adjacent non-lesion tissues were pooled respectively to generate single-cell suspensions with Adult Brain Dissociation Kit (Miltenyi Biotec). Myeloid cells were magnetically labeled with CD11b MicroBeads (Miltenyi Biotec), then isolated using a MACS separator.

### NanoString nCounter analysis

Myeloid cells isolated from spinal cord lesions and adjacent non-lesion tissues were lysed, respectively with modified chaotropic lysis buffer (QIAGEN). Whole-cell lysate from 45,000 cells was used for each hybridization reaction for the nCounter® mouse metabolic pathways panel (NanoString Technologies). nCounter® analysis was performed with the MAX/FLEX system at Georgetown University Genomics and Epigenomics Shared Resource. Normalized data were generated with nSolver 4.0 and then further analyzed on the Rosalind platform (https://www.rosalind.bio/).

### Immunostaining for demyelinated lesions

To prepare samples for immunohistochemistry, lesioned mice were anaesthetized with 3% isoflurane (Pivetal) before perfusion with cold 4% paraformaldehyde (PFA, Sigma-Aldrich) in PBS. Dissected spinal cord tissues were post-fixed at 4 °C for 30 min, then incubated in 30% sucrose solution (w/v) in 1X PBS at 4 °C overnight, before being embedded in O.C.T. Compound (Sakura) for freezing. Frozen tissues were cut into 12 µm sections with cryostat (Leica CM1860).

For immunostaining, cryo-sectioned tissues were dried for 1 h at room temperature (RT), washed with TBST (0.05% Tween 20 in 1X TBS) and subsequently with TBS, permeabilized for 15 min with permeabilization solution (1%Triton X-100 in 1X TBS), blocked for 1 h at RT with antibody diluent (10% serum and 0.25% Triton X-100 in 1X TBS), and then stained at 4 °C overnight with the following primary antibodies: rabbit anti-Olig2 (EMD Millipore, #AB9610, 1:300), mouse anti-APC (CC1, EMD Millipore, #OP80, 1:200), rabbit anti-Iba1 (FUJIFILM Wako, #019-19741, 1:400), mouse anti-iNOS (BD Biosciences, #610329, 1:100), chicken anti-Arginase-1 (Arg1, Millipore Sigma, #ABS535, 1:1000), rabbit anti-CYP1A1 (Proteintech, #13241-1-AP, 1:200), rabbit anti-CYP1B1 (Proteintech, #18505-1-AP, 1:50), mouse anti-AHR (Invitrogen, #MA1-514, 1:200), rat anti-P2RY12 (BioLegend, #848002, 1:100), rat anti-PDGFR-α (BD Biosciences,#558774, 1:500), mouse anti-O4 (R&D Systems, #MAB1326, 1:100), mouse anti-β-gal (Sigma-Aldrich, #G8021, 1:100), rat anti-CD3 (eBioscience, #14-0032-85, 1:100), rat anti-B220 (Invitrogen, #14-0452-81, 1:100), rat anti-CD11b (BioRad, #MCA74G, 1:100), rabbit anti-Sox9 (EMD Millipore, #AB5535, 1:1500), rat anti-CD68 (BioLegend, #137020, 1:100), rabbit anti-pS6 (Cell Signaling Technology, #5018, 1:1000). After incubation, slides were washed with TBST and TBS, and incubated with fluorescent secondary antibodies (Alexa Fluor™ 488, Alexa Fluor™ 594, Invitrogen, 1:500; Cy™3, Jackson ImmunoResearch,1:1000) and Hoechst 33342 (Invitrogen, 1:1000) at RT in dark for 1 h. The slides were further washed with TBST and TBS, then mounted with Fluoromount-G™ Mounting Medium (Invitrogen), and dried in dark at RT overnight. Stained slides were stored at 4 °C.

For Olig2 staining, antigen retrieval was performed before staining by incubating the slides with heated 1× Antigen Unmasking Solution (Vector laboratories) in glassware for 30 min. For staining with primary antibodies produced in mice, slides were incubated with MOM IgG blocking solution (Vector laboratories) for 1 h at RT before adding the primary antibodies. For β-gal staining, sectioned were permeabilized with cold methanol at −20 °C for 10 min, washed, then incubated with blocking buffer (10% serum, 1% BSA, and 0.25% Triton X-100 in 1X TBS) before primary antibody. For CC1 staining, the primary antibody was incubated at 4 °C for two nights. For conjugated antibodies, secondary antibody staining was skipped.

### Scanning electron microscopy and g-ratio

Focal demyelinated mice were injected with 1% neutral red (NR) to label lesions and perfused with a cold fixation buffer containing 2.5% glutaraldehyde and 1% paraformaldehyde in 0.2 M sodium cacodylate buffer (Electron microscopy sciences). Spinal cord lesions were dissected out and stored in fixative at 4 °C.

For sample processing, NR-labeled lesions were cut into small (<2 mm × 2 mm) pieces with a razor blade, washed in cold cacodylate buffer containing 2 mM calcium chloride, then incubated in the osmium solution for 1 h on ice. The tissue was then washed with ddH_2_O, incubated with 1% thiocarbohydrazide (TCH) solution (Ted Pella) for 20 min at RT, washed again with ddH_2_O, incubated with 2% osmium tetroxide for 30 min at RT, washed one more time with ddH_2_O, then incubated with 1% uranyl acetate at 4 °C overnight. Afterward, the tissue was washed in ddH_2_O and incubated in pre-heated lead aspartate solution in a 60 °C oven for 30 min. After incubation, lesion tissues were washed with ddH2O, and dehydrated with ice-cold freshly-prepared 50%, 70%, 85%, 95%, 100% ethanol solutions, and propylene oxide. The tissues were then embedded in resin and incubated in a 60 °C oven for 48 h.

For imaging acquisition, ultrathin sections (120 nm) containing spinal cord lesions were mounted in silicon wafers and observed with a Teneo LV FEG scanning electron microscope (FEI, Thermo Fisher Scientific). For optimal results, we used the optiplan mode (high-resolution) equipped with an in-lens T1 detector (Segmented A + B, working distance of 8 mm). Low-magnification images (600×) of the entire spinal cord section were first taken to delineate the lesioned site (ventral region/spinal cord), then we performed high magnification tile images with multiple captures of our regions of interest (10,000×) to ensure comprehensive coverage of the demyelinated lesioned area using 2 kV and 0.4 current landing voltage. For quantification, 2 mice (>200 remyelinated axons) from each group were measured for axon diameters and g-ratios (inner myelin diameter/outer myelin diameter) with MyelTracer^57^.

### Mass spectrometry for mouse lesions

Lesioned mice were injected with 1% neutral red 2 h before cold PBS perfusion. Neutral red-labeled lesions and adjacent non-lesion tissues were dissected on ice under a stereo microscope. Tissue samples were mixed with 500 μL of methanol containing the internal standard and homogenized for protein quantification assay with Pierce™ BCA Protein Assay Kit (ThermoFisher). Extracted supernatant from remaining homogenization was vacuum dried and derivatized. 1.5 µL of the derivatized solution was injected into an Agilent 7890B GC system (Santa Clara, CA, USA) that was coupled with a Pegasus HT TOF-MS (LECO Corporation, St. Joseph, MI, USA). Peak picking and alignments were performed using ChromaTof 4.7.2 (LECO Corporation). The BCA protein quantitation assay was used to normalize the peak areas. Mass spectra were compared to literature spectra available in the NIST database as well as the Fiehn library of compounds. The heatmap of analyzed metabolites was generated using Metabo Analyst. Statistical analysis of the absolute concentrations of PPA, IPA, and HPPA was performed by GraphPad Prism 9.

### RAW 264.7 cell culture and AKA treatment

The murine macrophage RAW 264.7 cell line was purchased at Georgetown University Tissue Culture and Biobanking Shared Resource. Cells were seeded at 5 × 10^4^ live cells per well on a 24-well plate in growth media (RPMI-1640 medium with heat-inactivated fetal bovine serum (FBS), GlutaMAX™ supplement, and antibiotic-antimycotic solution) and incubated at 37 °C with 5% CO_2_ for 2–3 days to obtain ~80% confluence. To prepare for in vitro treatment, PPA, IPA, and HPPA were dissolved at 1000× (250 mM), respectively in DMSO (Sigma-Aldrich). Cells were treated with 100 ng/mL lipopolysaccharides (LPS, Millipore Sigma), 250 μM PPA, 250 μM IPA, and 250 μM HPPA for 24 h. Volume-matched PBS and DMSO were used as vehicle controls.

### Microglia culture and AKA treatment

Microglia were MACS-purified with anti-CD11b microbeads (Miltenyi) from neonatal (P0–P3) cortices from Crl:CD(SD) rats and C57BL/6 mice. Cells were seeded at 100,000 live cells per well on poly-D-lysine hydrobromide (PDL, Millipore Sigma) coated 12 mm coverslips, and maintained in growth media (DMEM/F12 medium with heat-inactivated FBS, GlutaMAX™ supplement, and antibiotic-antimycotic solution) for a week. Cells were treated with 100 ng/mL LPS and 250 μM PPA + 250 μM IPA + 250 μM HPPA (or DMSO) for 24 h.

### Mixed glial culture and AKA treatment

The mixed glial culture was generated from the cortex of neonatal pups (P0–P3) from Crl:CD(SD) rats and C57BL/6 mice. Cells were plated on PDL-coated coverslips and incubated in growth media (DMEM/F12 medium with heat-inactivated FBS, GlutaMAX™ supplement, and antibiotic-antimycotic solution) for two weeks. To examine the effect of AKAs on oligodendrocyte differentiation, cells were supplied with fresh growth media containing 100 ng/mL LPS and 250 μM PPA + 250 μM IPA + 250 μM HPPA (or 0.3% DMSO) every other day for a week.

### Mixed glial culture and IL4I1 treatment

Mixed glia cultures were obtained from mouse (P3–P5) cortices and maintained in growth media (DMEM/F12 containing FBS, GlutaMAX™ supplement, and penicillin/streptomycin) for 2 weeks. To determine if IL4I1 mediates oligodendrocyte lineage cell function under an inflammatory environment, we treated mixed glial cells with 100 ng/mL LPS + 200 ng/mL recombinant mouse IL4I1, or PBS for 2 days in serum-free medium and fixed the cells after treatment for immunostaining to check oligodendrocyte differentiation.

### Primary oligodendrocyte cultures and AKA treatment

OPCs were purified from Sprague-Dawley rat pups (P6–P8) by immunopanning as previously described^58,59^. Briefly, cortical tissue was dissociated by papain digestion, then filtered through a Nitex mesh to obtain a mixed single-cell suspension. This suspension was incubated in two negative-selection plates coated with anti-RAN2 and anti-GALC antibodies to eliminate astrocytes and mature oligodendrocytes, respectively, then in a positive-selection plate coated with the anti-O4 antibody to select for OPCs. Adherent cells were trypsinized, cultured on glass coverslips sonicated for 1 h in ethanol, and coated with PDL hydrobromide. Using this method, we harvested 99.9% pure OPCs with less than 0.1% astrocyte and 0% microglia contamination. 10,000 OPCs were seeded per 12-mm coverslip. OPCs were first cultured in proliferation media (DMEM-Sato Base Growth Medium with Forskolin, CNTF, PDGF, and NT3) for 24 h, then switched to differentiation media (DMEM-Sato Base Growth Medium with Forskolin, CNTF, and T3) for the rest of the experiment. At three DIV, cells were treated with 250 μM PPA, 250 μM IPA, 250 μM HPPA, (or 0.1% DMSO) for 24 h.

### Primary oligodendrocyte cultures and IL4I1 treatment

OPC cultures for IL4I1 treatment were prepared from neonatal mouse pups (P3–P5) cortices MACS-sorted with anti-O4 microbeads (Miltenyi). OPCs were expanded in growth media (DMEM/F12 medium with N2, B27, penicillin/streptomycin, bovine serum albumin, PDGF and FGF) for 48 h, then treated with 200 ng/mL recombinant mouse IL4I1, or PBS in differentiation media (DMEM/F12 medium with N2, B27, penicillin/streptomycin, insulin and T3) for 24 h, followed by immunostaining analysis.

### Immunostaining for cell cultures

To prepare samples for immunocytochemistry, treated cells were washed with PBS and fixed with 4% PFA in PBS. Coverslips were then further washed with PBS before transferring into a new plate. Cells were permeabilized by incubating for 10 min with the permeabilization solution (0.1%Triton X-100 in PBS) and blocked with 10% serum in PBS for 1 h at RT. Coverslips were incubated overnight at 4 °C with primary antibodies including rabbit anti-Olig2 (EMD Millipore, #AB9610, 1:300), mouse anti-CC1 (EMD Millipore, #OP80, 1:200), rat anti-MBP (Millipore Sigma, #MAB386, 1:500), rabbit anti-Iba1 (FUJIFILM Wako, #019-19741, 1:400), mouse anti-iNOS (BD Biosciences, #610329, 1:100), rabbit anti-CYP1B1 (Proteintech, #18505-1-AP, 1:50), rat anti-PDGFR-α (BD Biosciences, #558774, #1:500), mouse anti-O4 (R&D Systems, #MAB1326, 1:100), rat anti-CD11b (BioRad, #MCA74G, 1:100), and rabbit anti-cleaved caspase 3 (Cell Signaling Technology, #9661, 1:100). For O4 staining, the primary antibody was provided to live cells before fixation. For CC1 staining, the primary antibody was incubated for 2 days at 4 °C. On the following day, coverslips were washed with PBST (0.05% Tween 20 in 1X PBS) before incubated with secondary antibodies (Alexa Fluor™ 488, Cy™3, Alexa Fluor™ 570, Alexa Fluor™ 594, Alexa Fluor™ 647) and Hoechst 33342 (Invitrogen, 1:1000) at RT in dark for 1 h. After washing with PBST, coverslips were mounted with Fluoromount-G™ Mounting Medium (Invitrogen), and dried in dark at RT overnight. Stained slides were stored at 4 °C. For primary oligodendrocyte cultures with AKA treatment, cells were permeabilized with 0.1% Triton X-100 in PBS for 3 min, then blocked with 5% donkey serum and 1% BSA in PBS for 30 min. Coverslips were stained with rat anti-MBP antibody (Abcam ab7349, 1:100) and mouse anti-O4 antibody (R&D MAB1326, 1:250), then with fluorescent secondary antibodies (Jackson ImmunoResearch). After staining, coverslips were fixed in Vectashield with DAPI.

### Imaging and quantification

For spinal cord lesions, slides were imaged with confocal microscopy (Zeiss LSM 800). Images were blinded with cage number and ear tag before quantification. Cells were counted with image J in the lesioned area defined by Hoechst staining, and then calculated as per mm^2^ or as percentages. The average of results from 2–6 spinal cord sections from each mouse was defined as *n* of one. For each group, *n* = 3–8 mice were used for statistics. For RAW 264.7 cells, microglia, and mixed glial cultures with AKA treatment were imaged with confocal microscopy (Zeiss LSM 800) and counted with image J. Results from five views of images in each condition (*n* = 3–4 repeats in total) were examined for statistics. For primary oligodendrocyte cultures with IL4I1 treatment, all views of images from 2 independent experiments were used for quantification. For primary oligodendrocyte cultures with AKA treatment, stained oligodendrocytes were imaged on a Nikon Ti2-E microscope with a Yokogawa CSU-X1 spinning disk and a Hamamatsu FusionBT camera. Cells were analyzed using NIS Elements software. Results from 3 independent experiments (3 rat pups) were used for statistics.

### ELISA

Conditioned media from AKAs-treated RAW 264.7 cell culture and microglia culture were collected, spun at 500 *g* for 5 min at 4 °C. Supernatant was aliquoted, and stored at −20 °C. Cytokine levels from the supernatant were detected with Mouse DuoSet ELISA kits (R&D Systems).

### RT-qPCR

Cells or tissues were collected into Trizol. RNA was isolated with Direct-zol RNA Miniprep Kits (Zymo Research), and reverse transcribed with iScript™ gDNA Clear cDNA Synthesis Kit (Bio-Rad). RT-qPCR reactions were set up with SYBR Green Supermix (Bio-Rad). Primers for target genes (*Il4i1*, *cyp1b1*) and housekeeping genes (*rpl13a*, *ppia*) were purchased from Bio-Rad. Expression levels (2^−ddCt^) were normalized to the control group (relative mRNA level = 1) of each independent experiment.

### Primers

Primers used for RT-qPCR were purchased from Bio-Rad. Primers used for transgenic mouse genotyping were synthesized by Eurofins Genomics. For IL4I1-tm1a, IL41I-tm1b, IL4I1-tm1c, and IL4I1-tm1d, the primers include Il4i1 5′ arm (5′-AGC TAC TCA AGG TGG TGA CCT-3’), Il4i1 3′ arm (5′-ACC TCA GCT CCC TCA CTA CAT-3′), and LAR3 (5′-CAA CGG GTT CTT CTG TTA GTC C-3′). For IL4I1-KO, primers were primer Neo3a (5′-GCA GCG CAT CGC CTT CTA TC-3′), primer 0770-29 (5′-GTG CTC ACT TCC TCT TTG CGA CT-3′), primer 0770-31 (5′-TTG AGA CCT TTC TTT CCG AGC AG-3′), and primer 0770-32 (5′-ACT GGG CTC TGG CTA AAC CTT G-3′). For LysM-Cre, the primers include oIMR3066 (5′-CCC AGA AAT GCC AGA TTA CG-3′), oIMR3067 (5′-CTT GGG CTG CCA GAA TTT CTC-3′), and oIMR3068 (5′-TTA CAG TCG GCC AGG CTG AC-3′). For TdTomato reporter line, the primers used were oIMR9020 (5′-AAG GGA GCT GCA GTG GAG TA-3′), oIMR9021 (5′-CCG AAA ATC TGT GGG AAG TC-3′), oIMR9103 (5′-GGC ATT AAA GCA GCG TAT CC-3′), and oIMR9105 (5′-CTG TTC CTG TAC GGC ATG G-3′).

### Data Analysis

All statistics were performed using GraphPad Prism 9. Data are graphed as mean ± SEM. Each point represents an individual value. Statistical significance is reported as not significant (ns) *P* > 0.05, *P ≤ 0.05, **P ≤ 0.01, ***P ≤ 0.001.

### Ethics

Human research recruitment was done at Johns Hopkins Multiple Sclerosis Center, upon Institutional Review Board approval. Written informed consent was obtained from the participants to participate in the study. The ethics oversight is driven by Johns Hopkins University, School of Medicine, US. All animals in this study were used following the Institutional Animal Care and Use Committee (IACUC) protocol at Georgetown University Division of Comparative Medicine.

### Reporting summary

Further information on research design is available in the Nature Research Reporting Summary linked to this article.

### Supplementary information

Supplementary information

Reporting Summary

Supplementary data 1

Supplementary data 2

Supplementary data 3

## Data Availability

All data are available in the main text or the supplementary materials. Any additional data that support the findings of this study are available from the corresponding author upon request.

## Code availability

All datasets were generated and processed with publicly available software. The software, software version, and websites were described in the “Methods” section.
