# Supplementary material for: Myeloid cell-associated aromatic amino acid metabolism facilitates CNS myelin regeneration
Source: NPJ Regen Med. 2024 Jan 2;9:1. doi: 10.1038/s41536-023-00345-9 (PMC10762216; doi:10.1038/s41536-023-00345-9)
Supplement: Supplementary file 1 — Supplementary information [file 41536_2023_345_MOESM1_ESM.pdf]

SUPPLEMENTARY FIGURES

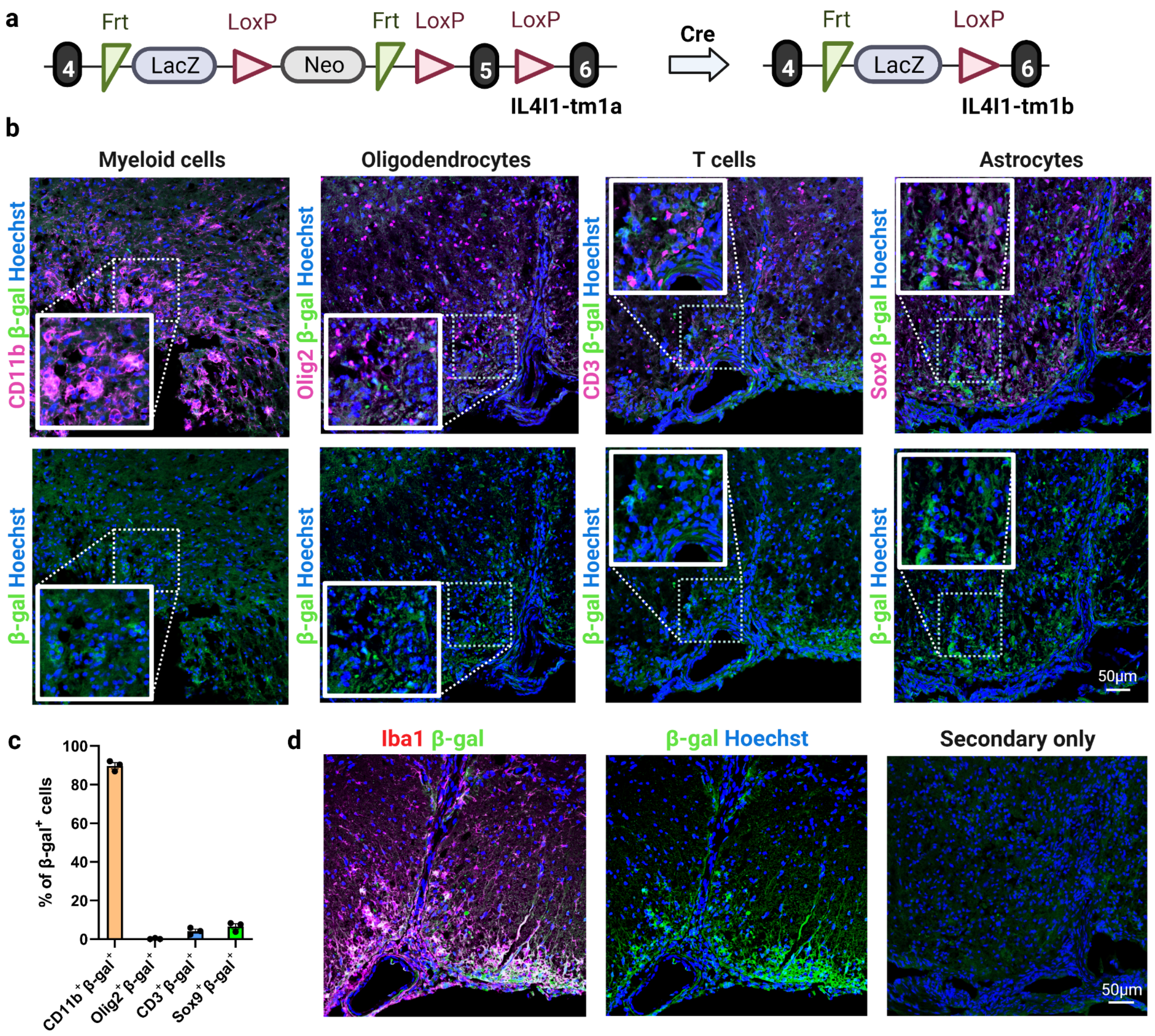

Supplementary Figure 1. IL4I1 expression pattern in demyelinated lesions. a, Schematic of generating IL4I1-tm1b reporter line. **b,** Representative images for immunostaining of *Il4i1*$^{tm1b/+}$ mouse lesions for LacZ reporter (β-gal), CD11b, Olig2, CD3, and Sox9. **c,** Quantification for CD11b^{+}β-gal^{+} myeloid cells, Olig2^{+}β-gal^{+} oligodendrocytes, CD3^{+}β-gal^{+} T cells, and Sox9^{+}β-gal^{+} astrocytes over all IL4I1-expressing (β-gal^{+}) cells in lesions (n=3 mice). **d,** Representative images of

immunostaining for LacZ reporter (β-gal), myeloid cells marker (Iba1), and secondary-only control in *Il4i1*$^{tm1b/+}$ mouse lesions. Bars represent the means with SEM. The scale bar represents 50 µm.

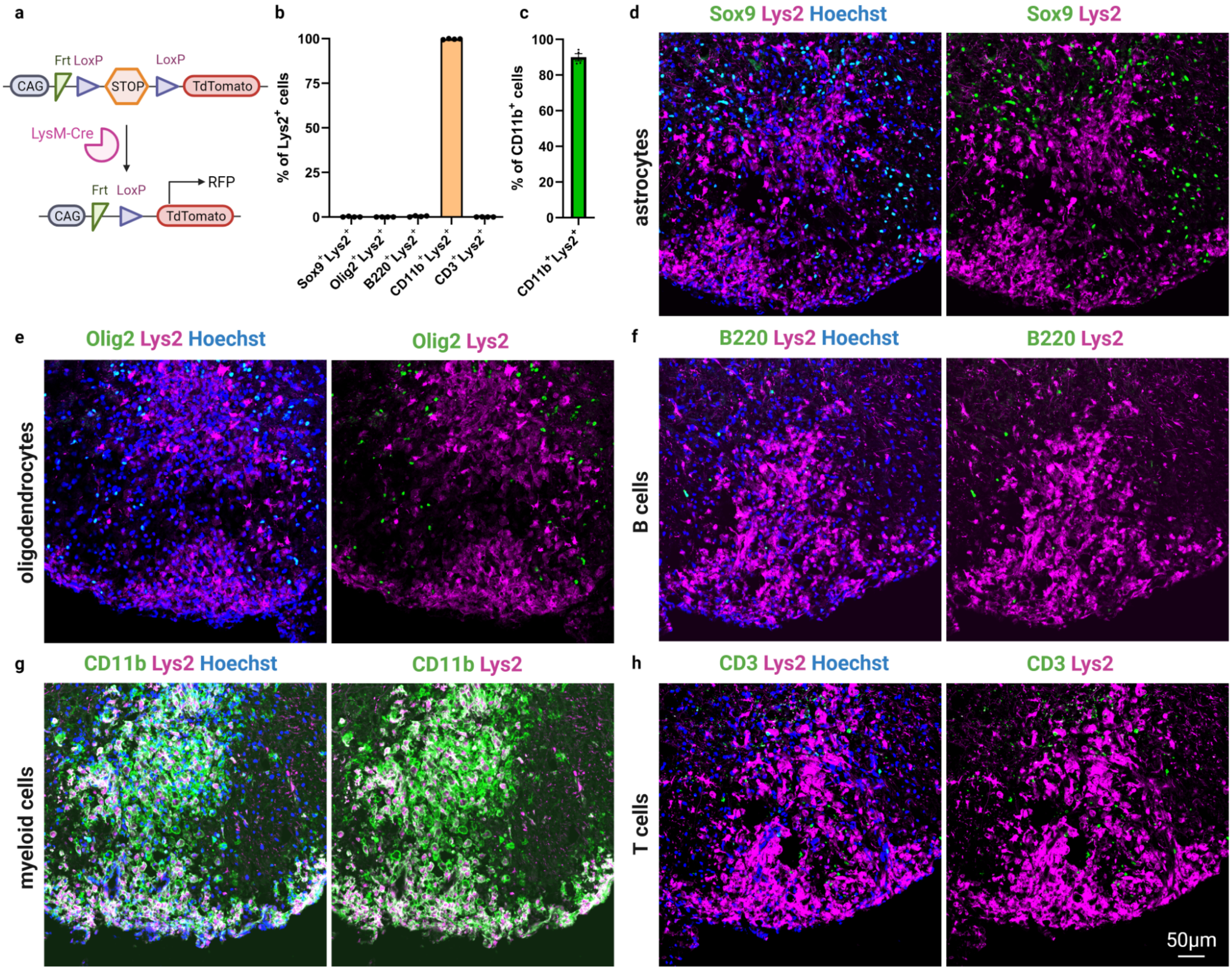

Supplementary Figure. 2. Characterization of LysM-Cre expression. a, Schematic of crossing LysM-Cre line with a ROSA reporter line, where Cre recombinase removes the stop codon and leads to the expression of endogenous red fluorescent protein (RFP). **b,** Quantification for Sox9$^+$Lys2$^+$ astrocytes, Olig2$^+$Lys2$^+$ oligodendrocytes, B220$^+$Lys2$^+$ B cells, CD11b$^+$Lys2$^+$ myeloid cells, and CD3$^+$Lys2$^+$ T cells over all Lys2$^+$ cells in lesions (n=4 mice). Bars represent the mean with SEM. **c,** Quantification for CD11b$^+$Lys2$^+$ cells over all CD11b$^+$ cells. **d-h,** Representative images for endogenous RFP that represents Lys2 expression, and immunostaining of Sox9, Olig2, B220, CD11b, and CD3. The scale bar = 50 μm.

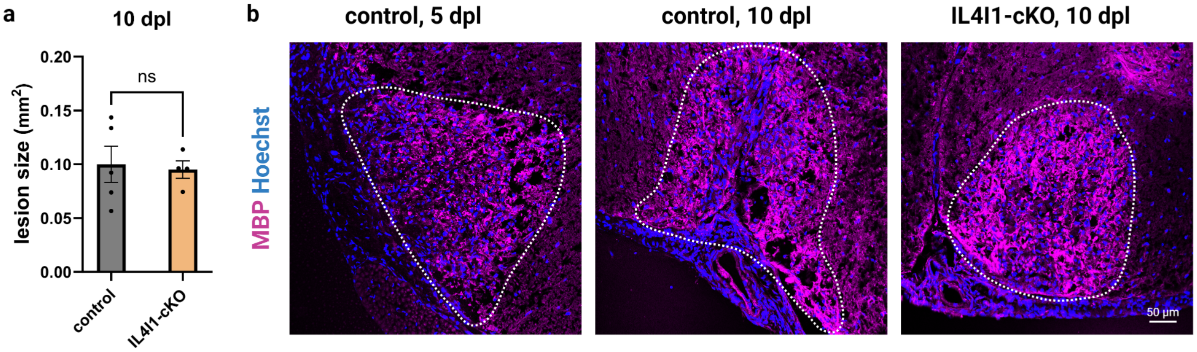

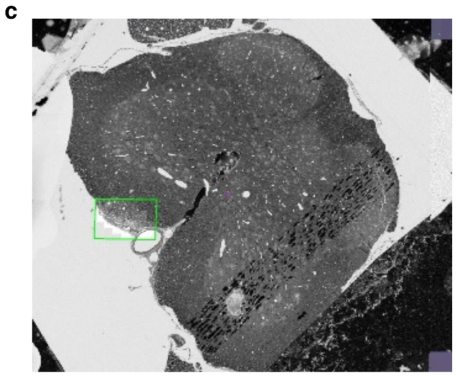

Supplementary Figure 3. Demyelinated lesion size of IL4I1-cKO and control mice. **a,** Quantification of the demyelinated lesion sizes in IL4I1-cKO and control mice at 10 dpl (n=4-5 mice, unpaired t-test). **b**, Representative images of MBP immunostaining in control animals at 5 dpl, which display more punctate and disconnected staining, indicative of myelin fragments, and in control and IL4I1-cKO mice at 10 dpl, which display smooth and connected staining, indicative of newly generated myelin. The scale bar = 50 μm. **c**, Representative low magnification image of a spinal cord section containing a focal lesion (green outline) under SEM. Once a lesion has been identified, the lesion is then imaged at high magnification. Bars represent the means with SEM. Each point represents an individual mouse. ns=non-significant.

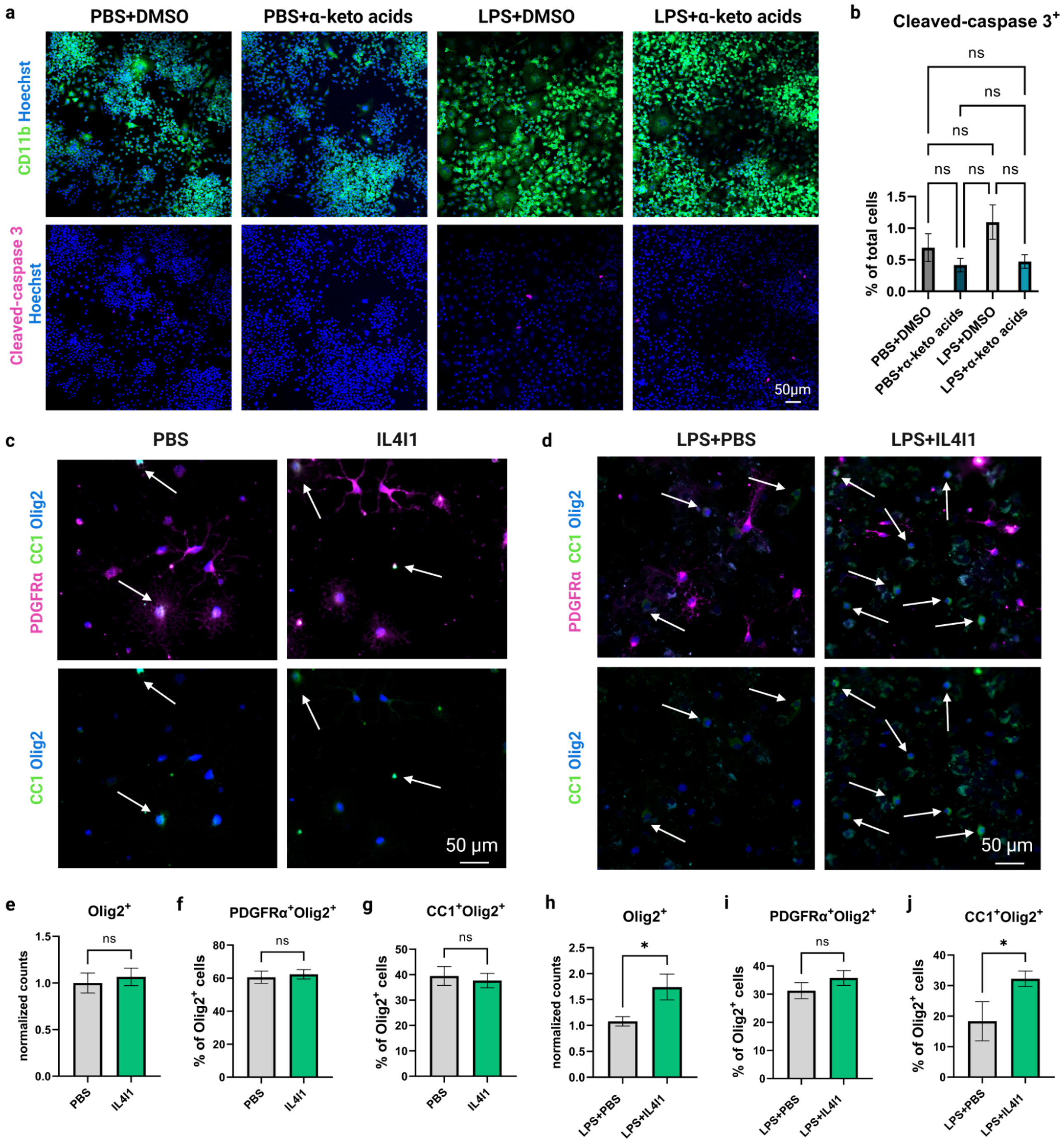

Supplementary Figure. 4. Viability test on AKAs-treated myeloid cells, and effect of IL4I1 on oligodendrocyte and mixed glial cultures. a, Representative image of

immunostaining for myeloid cells ($CD11b^+$) and apoptosis (cleaved-caspase 3^+) in RAW 264.7 cells treated with PBS+DMSO, PBS+AKAs, LPS+DMSO, and LPS+AKAs. **b,** Quantification of immunostaining for cleaved-caspase 3^+ cells over total cells (n=10 fields of view from two rounds of independent studies, Tukey's multiple comparisons test). **c,** Representative image of PDFGRα, CC1, and Olig2 staining in PBS or IL4I1-treated OPC cultures. **d,** Representative image of PDFGRα, CC1, and Olig2 staining in PBS or IL4I1-treated mixed glial cultures under LPS stimulation. **e-g,** Quantification of $Olig2^+$ oligodendrocyte linage cells (e), and the percentage of $Olig2^+PDGFRa^+$ immature oligodendrocytes (f) and $Olig2^+CC1^+$ mature oligodendrocytes (g) overall oligodendrocyte lineage cells. (n=15-19 fields of view from two independent studies, unpaired t-test.) **h-j,** Quantification of $Olig2^+$ oligodendrocyte lineage cells (h), and the percentage of $Olig2^+PDGFRa^+$ immature oligodendrocytes (i) and $Olig2^+CC1^+$ mature oligodendrocytes (j) over all $Olig2^+$ cells (n=5-19 fields of view from two independent studies, unpaired t-test). Arrows pointing at $CC1^+Olig2^+$ cells. The scale bar = 50 μm. Bars represent the means with SEM. *$P < 0.05$; ns=not significant.

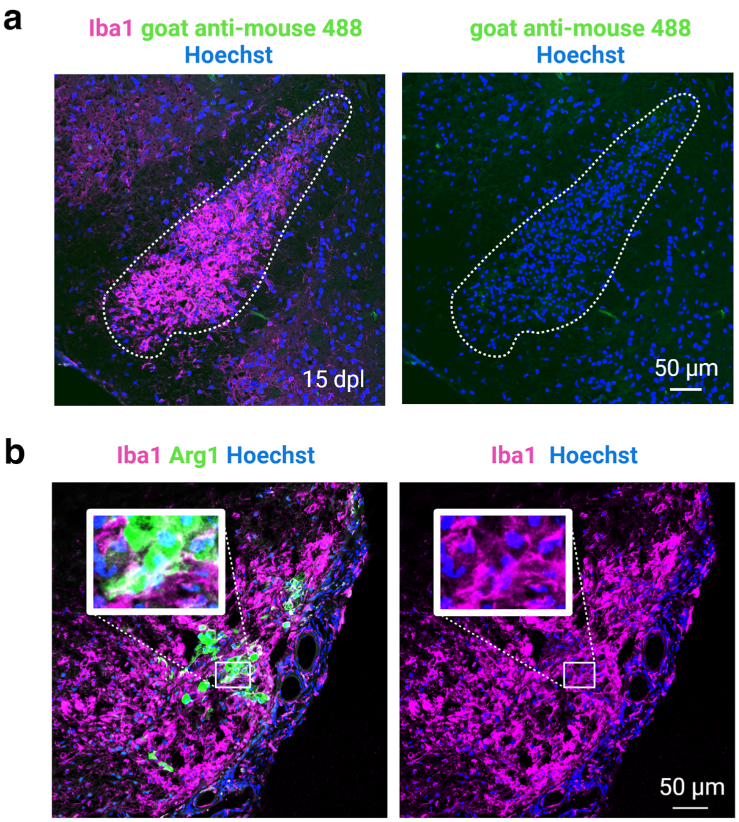

Supplementary Figure 5. iNOS and Arg1 staining of lesions. a, Image of secondary antibody-only control for iNOS staining. **b**, High-magnification representative image of Arg1 and Iba1 staining. The scale bar = 50 μm.

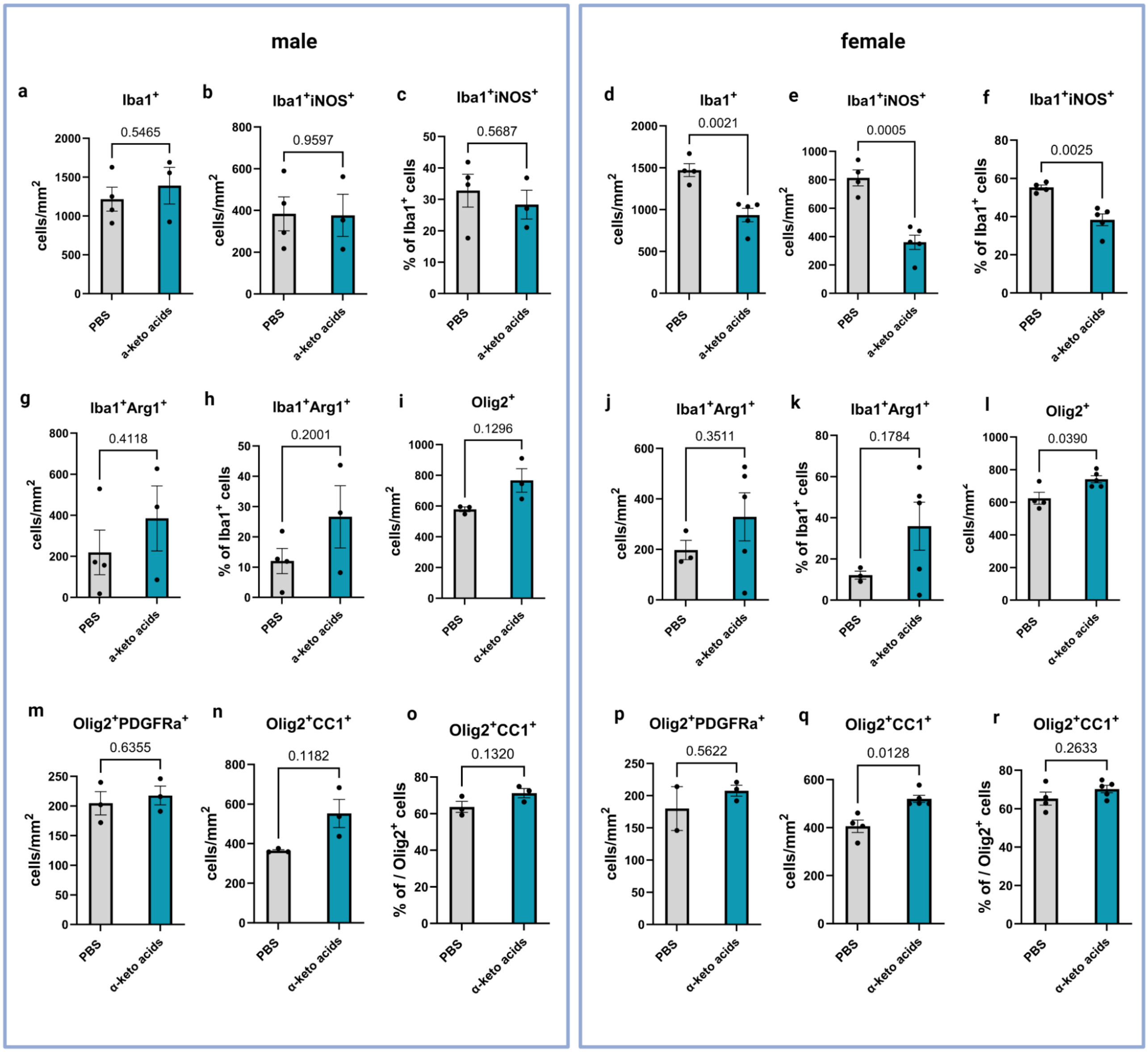

Supplementary Figure 6. The effect of AKAs on male and female lesioned mice. a-f, Quantification of Iba1$^+$ myeloid cells and Iba1$^+$iNOS$^+$ inflammatory myeloid cells in lesions from PBS or AKAs-treated male (n=3-4) and female mice (n=4-5, unpaired t-test). **g-l,** Quantification of Iba1$^+$Arg1$^+$ pro-regenerative myeloid cells and Olig2$^+$ oligodendrocyte lineage cells in lesions from PBS or AKAs-treated male (n=3-4) and

female mice (n=3-5, unpaired t-test). **m-r,** Quantification of Olig2^{+}PDGFRα^{+} OPCs, and Olig2^{+}CC1^{+} mature oligodendrocytes in lesions from PBS or AKAs-treated male (n=3) and female mice (n=2-5 mice, unpaired t-test). Bars represent the means with SEM. Each point represents an individual value.

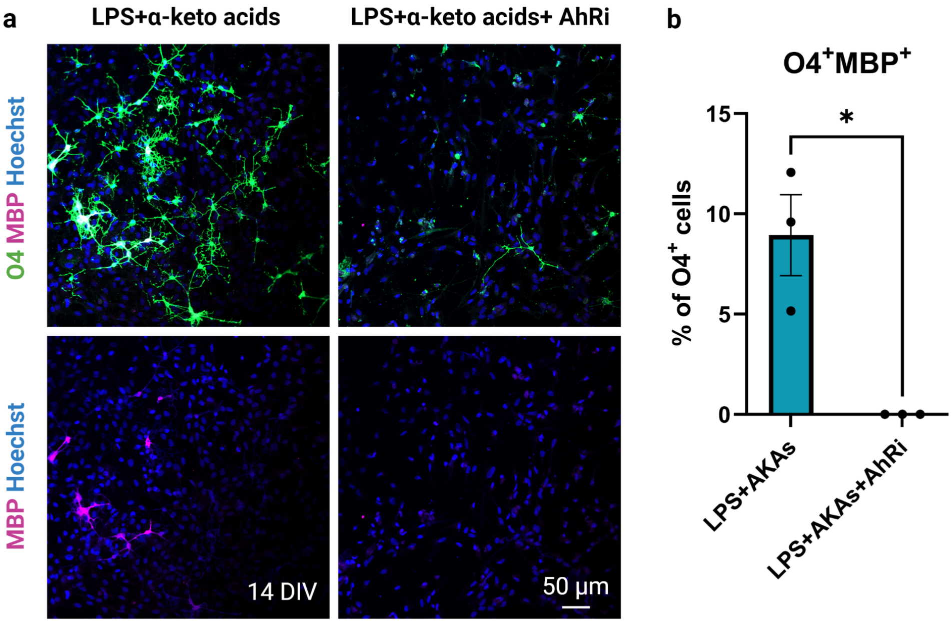

Supplementary Figure 7. AhR inhibitor treatment of mixed glial culture. a-b, Representative images, and quantification of O4^{+}MBP^{+} mature oligodendrocyte in mixed glial culture under LPS stimulation and AKAs treatment with or without AhR inhibitor (AhRi). The scale bar = 50 μm. *$P < 0.05$.
