# Supplementary material for: Myeloid cell-associated aromatic amino acid metabolism facilitates CNS myelin regeneration
Source: NPJ Regen Med. 2024 Jan 2;9:1. doi: 10.1038/s41536-023-00345-9 (PMC10762216; doi:10.1038/s41536-023-00345-9)
Supplement: Supplementary file 2 — Reporting Summary [file 41536_2023_345_MOESM2_ESM.pdf]

nature portfolio

Corresponding author(s): Dr Jeffrey Huang

Last updated by author(s): Sep 26, 2023

Reporting Summary

Nature Portfolio wishes to improve the reproducibility of the work that we publish. This form provides structure for consistency and transparency in reporting. For further information on Nature Portfolio policies, see our Editorial Policies and the Editorial Policy Checklist.

Statistics

For all statistical analyses, confirm that the following items are present in the figure legend, table legend, main text, or Methods section.

n/a	Confirmed	
☐	☒	The exact sample size (*n*) for each experimental group/condition, given as a discrete number and unit of measurement
☐	☒	A statement on whether measurements were taken from distinct samples or whether the same sample was measured repeatedly
☐	☒	The statistical test(s) used AND whether they are one- or two-sided<br>*Only common tests should be described solely by name; describe more complex techniques in the Methods section.*
☐	☒	A description of all covariates tested
☐	☒	A description of any assumptions or corrections, such as tests of normality and adjustment for multiple comparisons
☐	☒	A full description of the statistical parameters including central tendency (e.g. means) or other basic estimates (e.g. regression coefficient) AND variation (e.g. standard deviation) or associated estimates of uncertainty (e.g. confidence intervals)
☐	☒	For null hypothesis testing, the test statistic (e.g. *F*, *t*, *r*) with confidence intervals, effect sizes, degrees of freedom and *P* value noted<br>*Give P values as exact values whenever suitable.*
☒	☐	For Bayesian analysis, information on the choice of priors and Markov chain Monte Carlo settings
☒	☐	For hierarchical and complex designs, identification of the appropriate level for tests and full reporting of outcomes
☒	☐	Estimates of effect sizes (e.g. Cohen's *d*, Pearson's *r*), indicating how they were calculated

Our web collection on statistics for biologists contains articles on many of the points above.

Software and code

Policy information about availability of computer code

Data collection	ZEISS ZEN 3.3 (blue edition); FACSDiva software.
Data analysis	R v4.2.0; Rstudio v2022.07.2 Build 576; stats R package v4.2.0; impute R package v1.70.0; sva R package v3.44.0; gee R package v4.13-25; MatchIt R package v4.5.0; ggplot2 R package v3.4.0; nSolver 4.0; Rosalind platform (https://www.rosalind.bio/); MyelTracer; Metabo Analyst; GraphPad Prism 9; FCSExpress Version 7 (DeNovo software).

For manuscripts utilizing custom algorithms or software that are central to the research but not yet described in published literature, software must be made available to editors and reviewers. We strongly encourage code deposition in a community repository (e.g. GitHub). See the Nature Portfolio guidelines for submitting code & software for further information.

Data

Policy information about availability of data

All manuscripts must include a data availability statement. This statement should provide the following information, where applicable:

- Accession codes, unique identifiers, or web links for publicly available datasets
- A description of any restrictions on data availability
- For clinical datasets or third party data, please ensure that the statement adheres to our policy

The original data including Nanostring analysis read out and mass spectrometry data are available in the extended tables. All remaining raw data including genotyping gels, RT-qPCR readings, ELISA readings, immunostaining images and counts, and SEM images and counts are available in the lab records.

March 2021

Human research participants

Policy information about studies involving human research participants and Sex and Gender in Research.

Reporting on sex and gender	The study involved both females and males with a female:male ratio of 2-3:1, due to the disease being more prevalent in women. Participants self-reported their sex and gender identity. Sex was included in the analyses as a covariate to adjust for any effects it may have. The results may apply to both females and males.
Population characteristics	279 people with multiple sclerosis (MS) (205 females, 74 males; age range 18-72 years; 185 with relapsing-remitting MS [RRMS], 94 with progressive MS [PMS]) and 79 healthy controls without any known neurological disease (50 females, 29 males; age range 22-84 years).<br>162 people from the MS cohort were age-matched and used to compare metabolites between RRMS and PMS: 81 people with RRMS (65 females, 16 males; age range 26-70) and 81 people with PMS (53 females, 28 males; age range 26-72).<br>105 people from the MS cohort had available MRI data (78 females, 27 males; age range 18-72 years; 79 RRMS, 26 PMS) and were used for the association between brain volumetrics and metabolite concentrations.<br>Further details are provided in Extended Table 3.
Recruitment	Recruitment was done at Johns Hopkins Multiple Sclerosis Center, upon Institutional Review Board approval and after written informed consent.
Ethics oversight	Johns Hopkins University, School of Medicine, US

Note that full information on the approval of the study protocol must also be provided in the manuscript.

Field-specific reporting

Please select the one below that is the best fit for your research. If you are not sure, read the appropriate sections before making your selection.

☒ Life sciences ☐ Behavioural & social sciences ☐ Ecological, evolutionary & environmental sciences

For a reference copy of the document with all sections, see nature.com/documents/nr-reporting-summary-flat.pdf

Life sciences study design

All studies must disclose on these points even when the disclosure is negative.

Sample size	For lysolecithin-induced spinal cord focal demyelination mouse model, n=3-8 mice were used for each time point. For mass spectrometry analysis, n=10 mice from each genotype were used, because of the low level and big variance of the amino acid concentrations in the spinal cords. For Nanostring nCounter analysis, 3-5 dissected spinal cord lesions and adjacent non-lesion tissues were pooled respectively to generate one sample, n=2 samples from each group were used for the analysis. For scanning electron microscopy and g-ratio analysis, n= 202-502 axons from 2 mice were used in each group. For in vitro experiments, n=2-8 independent replicates.
Data exclusions	For figure 1g, one data point from the lesion group, which expressed too high level of Il4i1, was excluded after running the outlier test. No data were excluded from the analyses for the rest of the manuscript.
Replication	In vivo experiments were repeated 2-3 times using different animals to ensure reproducibility. In vitro experiments were repeated 2-8 times using cell line from different passages or primary culture from different animals to ensure reproducibility. All attempts at replication were successful.
Randomization	Mice for different treatment groups were randomly picked from litter mates. For example, if there were 4 male mice from one litter in a cage, 2 would be assigned to the control group, 2 would be assigned to the treatment group. All experiments ensure similar mice from each sex were used in each group.
Blinding	During sample collection, processing, imaging, and data analysis, each individual mouse were labeled as "cage number+ear tag" instead of genotype or treatment group to ensure the researcher is blinded and unbiased.

Reporting for specific materials, systems and methods

We require information from authors about some types of materials, experimental systems and methods used in many studies. Here, indicate whether each material, system or method listed is relevant to your study. If you are not sure if a list item applies to your research, read the appropriate section before selecting a response.

Materials & experimental systems

n/a	Involved in the study
☐	☒ Antibodies
☐	☒ Eukaryotic cell lines
☒	☐ Palaeontology and archaeology
☐	☒ Animals and other organisms
☐	☒ Clinical data
☐	☐ Dual use research of concern

Methods

n/a	Involved in the study
☒	☐ ChIP-seq
☐	☒ Flow cytometry
☐	☒ MRI-based neuroimaging

Antibodies

Antibodies used

rabbit anti-Olig2 (EMD Millipore, #AB9610), mouse anti-APC (CC1, EMD Millipore, #OP80), rabbit anti-Iba1 (FUJIFILM Wako, #019-19741), mouse anti-iNOS (BD Biosciences, #610329), chicken anti-Arginase-1 (Arg1, Millipore Sigma, #ABS535), rabbit anti-CYP1A1 (Proteintech, #13241-1-AP), rabbit anti-CYP1B1 (Proteintech, #18505-1-AP), mouse anti-AHR (Invitrogen, #MA1-514), rat anti-P2RY12 (BioLegend, #848002), rat anti-PDGFR-α (BD Biosciences, #558774), mouse anti-O4 (R&D Systems, #MAB1326), mouse anti-β-gal (Sigma-Aldrich, #G8021), rat anti-CD3 (eBioscience, #14-0032-85), rat anti-CD11b (BioRad, #MCA74G), rabbit anti-Sox9 (EMD Millipore, #AB5535), rat anti-CD68 (BioLegend, #137020), rabbit anti-pS6 (Cell Signaling Technology, #5018). rat anti-MBP (Millipore Sigma, #MAB386), rat anti-PDGFR-α (BD Biosciences, #558774), rabbit anti-cleaved caspase 3 (Cell Signaling Technology, #9661). rat anti-MBP antibody (Abcam, #ab7349).

Validation

All antibodies used in this study are commercially available and validated by the manufacturer. https://www.emdmillipore.com/US/en/product/Anti-Olig-2-Antibody,MM_NF-AB9610#anchor_COA; https://www.emdmillipore.com/US/en/product/Anti-APC-Ab-7-Mouse-mAb-CC-1,EMD_BIO-OP80#anchor_COA; https://www.rndsystems.com/datasheet/coa; https://www.bdbiosciences.com/en-us/products/reagents/flow-cytometry-reagents; https://www.ptglab.com/products/CYP1A1-Antibody-13241-1-AP.htm; https://www.abcam.com/myelin-basic-protein-antibody-12-ab7349.html; https://www.emdmillipore.com/US/en/life-science-research/antibodies-assays/antibodies-overview/Antibody-Development-and-Validation/cFOb.qB.8McAAAFOb64qQvSS,nav; https://www.thermofisher.com/us/en/home/life-science/antibodies/invitrogen-antibody-validation.html?icid=ab-search-learning-ab-validation; https://www.bio-rad-antibodies.com/our-guarantees-to-you.html; https://www.cellsignal.com/about-us/our-approach-process/cst-antibody-performance-guarantee; https://www.biolegend.com/en-us/quality/quality-control.

Eukaryotic cell lines

Policy information about cell lines and Sex and Gender in Research

Cell line source(s)

Murine macrophages RAW 264.7 cell line was provided by Georgetown University Tissue Culture and Biobanking Shared Resource.

Authentication

Murine macrophages RAW 264.7 cell line was not authenticated.

Mycoplasma contamination

Cell line was tested negative for mycoplasma.

Commonly misidentified lines
(See ICLAC register)

N/A

Animals and other research organisms

Policy information about studies involving animals; ARRIVE guidelines recommended for reporting animal research, and Sex and Gender in Research

Laboratory animals

C57BL/6 mice (RRID: IMSR_JAX:000664), ACTBFLPe mice (RRID: IMSR_JAX:005703), and LysMcre knock-in/knock-out mice (RRID: IMSR_JAX:004781) were purchased from the Jackson Laboratory. IL4I1-KO (Il4i1-/-) mice (RRID: MMRRC_011726-UCD) were purchased from Mutant Mouse Resource & Research Centers (MMRRC). IL4I1-tm1a (MGI: 4432453) and IL4I1-tm1b (MGI: 6120692) mice were purchased from European Mouse Mutant Archive (EMMA). Timed pregnant Crl:CD(SD) rats (RRID: RGD_734476) were purchased from Charles River. Mice of both sexes were used in this study. All above animals were housed at Georgetown University Division of Comparative Medicine following the IACUC protocol and maintained on a 12-hour-light/12-hour-dark cycle with food and water ad libitum. Sprague-Dawley rats used to prepare OPC cultures for AKAs treatment were shipped as moms and P5 pups from Charles River, and housed at the vivarium in the John Edward Porter Neuroscience Research Center (PNRC) at NINDS.

Wild animals

No wild animals were used in this study.

Reporting on sex

For the experiments in this study, mice/rats of both sexes were used.

Field-collected samples

The study did not involve samples collected from the field.

Ethics oversight

All animals were used following the IACUC protocol at Georgetown University Division of Comparative Medicine.

Note that full information on the approval of the study protocol must also be provided in the manuscript.

Clinical data

Policy information about clinical studies

All manuscripts should comply with the ICMJE guidelines for publication of clinical research and a completed CONSORT checklist must be included with all submissions.

Clinical trial registration	N/A
Study protocol	N/A
Data collection	Research visits (including blood draws) and data collection were performed at the Johns Hopkins Multiple Sclerosis Center; People were recruited from September 2003 till November 2018.
Outcomes	The primary outcome of the human cohort was to compare the concentration of metabolites of interest in the serum of people with MS and healthy controls, as well as between the different MS subtypes. Metabolomics analyses were performed at Metabolon (Durham, NC), using liquid and gas chromatography with mass spectrometry. Associations between metabolite levels and imaging brain outcomes were explored.

Dual use research of concern

Policy information about dual use research of concern

Hazards

Could the accidental, deliberate or reckless misuse of agents or technologies generated in the work, or the application of information presented in the manuscript, pose a threat to:

No	Yes	
☒	☐	Public health
☒	☐	National security
☒	☐	Crops and/or livestock
☒	☐	Ecosystems
☒	☐	Any other significant area

Experiments of concern

Does the work involve any of these experiments of concern:

No	Yes	
☒	☐	Demonstrate how to render a vaccine ineffective
☒	☐	Confer resistance to therapeutically useful antibiotics or antiviral agents
☒	☐	Enhance the virulence of a pathogen or render a nonpathogen virulent
☒	☐	Increase transmissibility of a pathogen
☒	☐	Alter the host range of a pathogen
☒	☐	Enable evasion of diagnostic/detection modalities
☒	☐	Enable the weaponization of a biological agent or toxin
☒	☐	Any other potentially harmful combination of experiments and agents

Flow Cytometry

Plots

Confirm that:

☐ The axis labels state the marker and fluorochrome used (e.g. CD4-FITC).

☐ The axis scales are clearly visible. Include numbers along axes only for bottom left plot of group (a 'group' is an analysis of identical markers).

☐ All plots are contour plots with outliers or pseudocolor plots.

☐ A numerical value for number of cells or percentage (with statistics) is provided.

Methodology

Sample preparation	*Describe the sample preparation, detailing the biological source of the cells and any tissue processing steps used.*
Instrument	*Identify the instrument used for data collection, specifying make and model number.*

Software	*Describe the software used to collect and analyze the flow cytometry data. For custom code that has been deposited into a community repository, provide accession details.*
Cell population abundance	*Describe the abundance of the relevant cell populations within post-sort fractions, providing details on the purity of the samples and how it was determined.*
Gating strategy	*Describe the gating strategy used for all relevant experiments, specifying the preliminary FSC/SSC gates of the starting cell population, indicating where boundaries between "positive" and "negative" staining cell populations are defined.*

☐ Tick this box to confirm that a figure exemplifying the gating strategy is provided in the Supplementary Information.

Magnetic resonance imaging

Experimental design

Design type	Cross-sectional in vivo MRI
Design specifications	One session per subject
Behavioral performance measures	None

Acquisition

Imaging type(s)	Structural
Field strength	3-tesla
Sequence & imaging parameters	Three-dimensional (3D) fluid-attenuated inversion recovery (FLAIR; 1mm×1mm×1mm; echo time (TE): 300 ms; repetition time (TR): 4.8s; inversion time (TI): 1.6s; SENSE factor: 2.6; averages: 1); T2-weighted dual-echo turbo spin echo (DE-TSE; acquired resolution: 0.8mm×0.8mm×3mm; TE(s): 11/100 ms; TR: 3450ms; SENSE factor: 2; averages: 1); and 3D magnetization prepared rapid acquisition of gradient echoes (MPRAGE; acquired resolution: 1mm×1mm×1 mm; TE: 3.6ms; TR: 7.5ms; TI: 900ms; flip angle: 9°; SENSE factor: 2; averages: 1)
Area of acquisition	Whole brain

Diffusion MRI ☐ Used ☒ Not used

Preprocessing

Preprocessing software	Preprocessing was completed using a custom pipeline based in Python. MRI images were corrected for intensity bias and co-registered to the MNI-152 atlas using the ANTs software package. Images were harmonized between different acquisitions using DeepHarmony. Whole brain segmentation was performed on harmonized images using SLANT-CRUISE. Briefly, the images are segmented using the SLANT deep learning algorithm and corrected to be consistent with cortical reconstruction using CRUISE, similar to Multi-Atlas CRUISE. In addition to whole brain, cortical gray matter, cerebral white matter, deep gray matter (including thalamic), and lesion volumes were computed. Volumes were normalized by intracranial volume , to account for differences in head size. Longitudinal ComBat was used to correct volumes for scanner changes.
Normalization	Subjects were registered to the MNI-152 (ICBM-152 2009c) atlas using a rigid transform. Intra-subject images were also co-registered with rigid transforms.
Normalization template	MNI-152 (ICBM-152 2009c)
Noise and artifact removal	The presence of artifacts was assessed by visual inspection
Volume censoring	N/A

Statistical modeling & inference

Model type and settings	Multivariable linear regression models were used with brain substructural volumes as the dependent variables and individual metabolite levels, sex, age and MS type as the independet variables.
Effect(s) tested	Phenylpyruvate, the ratio of phenylpyruvate to phenylalanine, 4-hydroxyphenylpyruvate and the ratio of 4-hydroxyphenylpyruvate to tyrosine.

Specify type of analysis: ☐ Whole brain ☐ ROI-based ☒ Both

Anatomical location(s)	Whole brain segmentation and sub-structure segmentation was performed on harmonized images using SLANT-CRUISE. Briefly, the images are segmented using the SLANT deep learning algorithm and corrected to be consistent with cortical reconstruction using CRUISE, similar to Multi-Atlas CRUISE.
Statistic type for inference (See Eklund et al. 2016)	N/A

Correction | N/A

Models & analysis

n/a	Involved in the study
☒	☐ Functional and/or effective connectivity
☒	☐ Graph analysis
☐	☒ Multivariate modeling or predictive analysis

Multivariate modeling and predictive analysis | Dependent variables included fractions of whole brain, cortical GM, cerebral WM, subcortical GM and T2 lesion volumes. Independent variables included phenylpyruvate, the ratio of phenylpyruvate to phenylalanine, 4-hydroxyphenylpyruvate and the ratio of 4-hydroxyphenylpyruvate to tyrosine. Covariates in all models included sex, age and MS subtype (RRMS, PMS).
